# Vascular Neuroembryology: The Genesis of the Intracranial Arterial System and the Circle of Willis

**DOI:** 10.3390/life16071153

**Published:** 2026-07-13

**Authors:** Karl Jacobs, Chelsey Marjana Linnenbank, Rene Van Den Berg, Geerling Langenbach, Karel de Bree, Jaco Hagoort, Berrie Meijer, Roelof Jan Oostra, Frank Lobbezoo, Victor Volovici, Bernadette Simone de Bakker

**Affiliations:** 1 Department of Oral Pain and Dysfunction, Academic Centre for Dentistry Amsterdam (ACTA), University of Amsterdam and Vrije Universiteit Amsterdam, 1081 LA Amsterdam, The Netherlands; g.langenbach@acta.nl (G.L.); f.lobbezoo@acta.nl (F.L.); 2 Department of Medical Biology, Section Clinical Anatomy & Embryology, Amsterdam UMC Location AMC, University of Amsterdam, 1105 AZ Amsterdam, The Netherlands; c.m.linnenbank@lumc.nl (C.M.L.); j.hagoort@amsterdamumc.nl (J.H.); r.j.oostra@amsterdamumc.nl (R.J.O.); 3 Amsterdam Reproduction and Development Research Institute, 1105 AZ Amsterdam, The Netherlands; b.s.debakker@amsterdamumc.nl; 4 Department of Human Genetics, Leiden University Medical Centre (LUMC), 2300 RC Leiden, The Netherlands; 5 Department Radiology and Nuclear Medicine, Amsterdam UMC Location AMC, University of Amsterdam, 1105 AZ Amsterdam, The Netherlands; r.vdberg@amsterdamumc.nl; 6 Department of Neurosurgery, Radboud University Medical Center, 6525 GA Nijmegen, The Netherlands; karel.debree@radboudumc.nl; 7 Department of Gastroenterology and Hepatology, Dijklander Hospital, 1624 NP Hoorn, The Netherlands; b.meijer@dijklander.nl; 8 Department of Orofacial Pain and Jaw Function, Faculty of Odontology, Malmö University, 214 21 Malmö, Sweden; 9 Department of Neurosurgery, Erasmus MC Rotterdam, 3015 GD Rotterdam, The Netherlands; v.volovici@erasmusmc.nl; 10 Center for Complex Microvascular Surgery, Erasmus MC University Medical Center, 3015 GD Rotterdam, The Netherlands; 11 Department of Obstetrics and Gynecology, Amsterdam UMC Location AMC, 1105 AZ Amsterdam, The Netherlands; 12 Department of Pediatric Surgery, Erasmus MC—Sophia Children’s Hospital, University Medical Center Rotterdam, 3015 GD Rotterdam, The Netherlands

**Keywords:** cerebrovascular development, neurovascular embryology, vascular remodeling, circle of Willis, 3D reconstruction, stroke, congenital vascular malformation

## Abstract

Cerebrovascular disorders, encompassing conditions such as stroke, aneurysms, and arteriovenous malformations, are often associated with developmental malformations of the intracranial arterial system, including the circle of Willis. The circle of Willis plays a crucial role in establishing a collateral vascular network essential for ensuring adequate cerebral blood supply. However, the intricate process of human intracranial vascular development, known as vascular neuroembryology, remains complex and incompletely understood. The diverse array of anatomical vascular variations observed in clinical practice is a direct outcome of this developmental process. We present a chronological overview of human neurovascular embryology using stage-resolved three-dimensional reconstructions of the developing intracranial arterial system. Histological sections of 26 human embryos ranging from Carnegie stages (CS) 11 to 23 were digitally reconstructed and manually segmented in Amira software (versions 5.3–5.6; Thermo Fisher Scientific, Waltham, MA, USA) to generate detailed 3D models of the cranial vasculature and adjacent anatomical structures, including the neural tube, cranial nerves, eye, and ear. Sequential 3D reconstruction revealed progressive remodeling of the primitive cranial vasculature and key developmental transitions underlying formation of the circle of Willis. By integrating these developmental observations with existing embryological literature, we refine current models of neurovascular development, with particular emphasis on the ophthalmic and trigeminal arterial systems. These findings provide an updated developmental framework for interpreting normal and variant intracranial vascular anatomy encountered in clinical practice.

## 1. Introduction

### 1.1. The Circle of Willis

Cerebrovascular disorders, encompassing conditions such as stroke, aneurysms, and arteriovenous malformations, are often seen in association with developmental malformations in the intracranial arterial system, including the circle of Willis (CoW). Stroke remains the second leading cause of mortality worldwide and the fifth leading cause of death in the United States [1,2]. Understanding developmental determinants of anatomical variation in the CoW is therefore of paramount importance for clinicians and researchers worldwide. A fully developed CoW is critical for establishing a collateral vascular network that ensures adequate blood supply to the brain in case of local vascular impairment. However, the prevalence of anatomical variants within the CoW, including fenestrated, duplicated, hypoplastic, or absent vessels, is estimated to be as high as 68% in the general population [3,4]. Developmentally determined variations of the CoW have been implicated as modifiers of cerebrovascular vulnerability, including ischemic stroke, aneurysm formation, and arteriovenous malformations [5]. These variations serve as a guidance for various neurointerventional and diagnostic procedures. Several population-based studies have demonstrated that incomplete configurations of the CoW are associated with an increased susceptibility to ischemic stroke, an effect that is thought to reflect reduced collateral capacity rather than direct causation [6]. In one of these studies (*n* = 976), an incomplete anterior CoW was associated with a nearly threefold increased risk of future anterior circulation ischemic stroke (hazard ratio 2.8, 95% CI 1.3–6.3). Additionally, a meta-analysis involving 2718 participants showed that individuals with CoW variation were approximately 1.4 times more likely to develop ischemic stroke, consistent with a positive association [2].

Despite these well-documented clinical associations, the embryological mechanisms and precise developmental time windows that give rise to such anatomical variation of the CoW remain incompletely understood. In particular, the timing of arterial appearance, regression, remodeling, and establishment of the definitive cerebral circulation during human embryogenesis remains incompletely resolved. Consequently, it is unclear at which stages developmental deviations may predispose to the diverse configurations observed in adult cerebrovascular anatomy. Beyond ischemic stroke, CoW variants such as fenestrations and duplications are also associated with an increased risk of saccular brain aneurysm formation [7,8]. The concept of geometric risk for atherosclerosis also highlights the relationship between arterial geometry and stroke risk, as atherosclerotic plaques typically arise in regions of low or oscillatory wall shear stress [9,10]. Awareness of arterial variants is furthermore crucial for medico-surgical interventions. For example, distinguishing congenital absence of the internal carotid artery (ICA) or ICA hypoplasia from acquired stenosis is essential [11]. An aberrant ICA can mimic several medical and surgical conditions, including glomus tumor, dehiscent jugular bulb, cholesterol granuloma, and petrous carotid aneurysm [12,13]. Understanding these neurovascular anomalies is therefore indispensable for accurate clinical evaluation and management.

#### 1.1.1. Embryological Development of the Circle of Willis

The process of cranial arterial development starts early in the embryonic period, at Carnegie stage 11 (CS11, 2.5 mm CRL, 23–26 days post fertilization) [14]. A cascade of biological processes, including precursor cell migration, de novo vessel formation (vasculogenesis), and remodeling of primitive channels (angiogenesis), collectively drive the development of the cranial vascular tree [15]. Throughout embryogenesis, which spans the initial eight weeks post fertilization, the cerebral circulation undergoes continuous adaptations to secure an adequate blood supply to the developing brain [16]. Between CS11 and CS20 (23–56 days), the mature vascular pattern of the CoW begins to manifest and becomes clearly recognizable toward the end of the embryonic period (CS20, ~15.9 mm, 51–53 days). Further anatomical maturation continues throughout the fetal period.

The developing brain has a high metabolic demand and is highly sensitive to ischemia [17]. To maintain sufficient perfusion, the cerebral circulation relies on an extensive collateral network and the precise regulation of vasodilation and vasoconstriction [18,19]. The collateral circulatory system, which secures the brain’s blood supply, can be divided into primary and secondary routes. The CoW forms the primary collateral pathway, whereas secondary collateral routes include leptomeningeal anastomoses and extracranial/intracranial arterial connections that may provide additional perfusion during arterial occlusion [20]. Structurally, the CoW is a ring-like arterial configuration that links the anterior and posterior circulations and interconnects the principal cerebral arteries located in close proximity [17,20,21]. Following normal development, the anterior circulation is supplied by the bilateral ICAs, while the posterior circulation derives from the basilar artery (BA), itself formed by the fusion of the vertebral arteries. Ultimately, the development of the cerebral vascular tree results in a somewhat unique configuration for each individual [15].

#### 1.1.2. Aim of This Study

The aim of this study is to provide a comprehensive, state-of-the-art overview of a reconstruction-based, stage-resolved analysis of neurovascular development, with a particular focus on the genesis of the CoW. Utilizing histological sections of human embryos from the 3D Embryo Atlas [14], the most comprehensive digital embryonic database to date, we generated detailed interactive 3D reconstructions of neurovascular development. These reconstructions illustrate the sequential formation of the CoW and identify critical developmental transitions that may underlie anatomical variation and congenital vascular configurations. By cross-referencing our findings with the existing literature, we refine current models of neurovascular development by identifying stage-specific transitions underlying circle of Willis formation, thereby providing a developmental framework for interpreting variability in adult cerebral arterial anatomy.

## 2. Materials and Methods

### 2.1. Experimental Research Methods

#### 2.1.1. Human Specimens

Specimens used to reconstruct the embryological evolution of the intracranial arteries were extracted from the 3D Embryo Atlas, a digitized collection of serially sectioned human embryos covering Carnegie stages (CS) 7–23 [14]. For the present study, we analyzed a subset of 26 embryos between CS11 and CS23 (13 stages, two specimens per stage), representing the critical developmental window for intracranial arterial formation. For reconstruction and visualization purposes, the specimen demonstrating the best preservation quality and vascular visibility was selected, while the second specimen was used as a comparative reference during assessment of intracranial arterial development. Overall, the principal vascular patterns were consistent between paired specimens of the same Carnegie stage. Most specimens originated from the historical Carnegie collection (Silver Spring, MD, USA). They were collected between 1910 and 1975 following hysterectomy (*n* = 8), miscarriage (*n* = 5), abortion (*n* = 4), or with no information available (*n* = 9). In addition, two (*n* = 2) specimens were sourced from the Boyd Collection at the University of Cambridge, UK, and one (*n* = 1) specimen from the Department of Medical Biology, Amsterdam UMC, the Netherlands [14] (Supplementary Table S1). The specimens were fixed, embedded, sectioned, and stained using histological staining methods (mainly hematoxylin–eosin). The serial histological sections were available as a duplicate series covering CS11–23 (13 stages), which corresponds to the first five weeks of intracranial vascular formation. The embryos were classified according to Streeter’s original classification [22] (Streeter, 1942) and the modified version by O’Rahilly and Müller [23]. The embryonic specimens used in this study are part of the previously described 3D Embryo Atlas dataset [14]. As this study exclusively involved analysis of existing digital reconstructions of historical specimens, no additional ethical approval was required.

#### 2.1.2. Image Acquisition and Processing

Image acquisition and preprocessing were performed as described in detail previously [14]. In short, digital images of the embryonic sections were acquired using a brightfield microscope equipped with a motorized XY-stage. Capturing and stitching of tiled images were performed, including flat-field correction.

In addition, a macro-imaging setup was used for specimen imaging. As a final preprocessing step, images were subjected to color-to-grayscale conversion, resolution reduction, cropping, and contrast enhancement.

#### 2.1.3. Alignment, Segmentation, and Visualization in Amira

The 3D Embryo Atlas forms the basis for the 3D models used in this study. The reconstruction process of the original models has been described in detail previously [14]. In short, Amira software (versions 5.3–5.6; Thermo Fisher Scientific, Waltham, MA, USA) was used for alignment of image stacks, segmentation, and visualization.

Three-dimensional structures were manually outlined using the Segmentation Editor in combination with a Bamboo tablet and pen (Wacom Co., Ltd., Kazo, Saitama, Japan) by trained (bio)medical students under supervision of the last author. This approach resulted in 3D segmentations with a volumetric inter-observer variability of 0.3% in large and simple structures (e.g., neural tube) and 2% in more detailed structures (e.g., vessels) [14].

Visualization was achieved by converting segmentation label files into triangulated surface files using the SurfaceGen function. The resulting datasets were optimized through post-processing steps, including label resolution reduction (Resample), interpolation (InterpolateLabels), triangle reduction (Simplify), and surface smoothing (SmoothSurface), to enable efficient modeling and visualization.

For visualization purposes, the detailed Amira models were subsequently transformed into smooth and informative representations using knowledge-driven modeling in Blender software (version 2.51–2.76; Blender Foundation, Amsterdam, The Netherlands). Blender files were exported as Autodesk FBX (.fbx) files.

For the present study, we focused on intracranial arteries at each developmental stage. To this end, the cranial arteries were segmented again in greater detail using the same segmentation and visualization workflow in Amira software (versions 6.5–2024.2; Thermo Fisher Scientific, Waltham, MA, USA).

#### 2.1.4. Interactive 3D-PDF

Simplified and smoothed models of the cranial arteries (Amira surface files, .surf) were converted to .u3d files using Fiji (version 2.16/ImageJ 1.54p). These files were subsequently processed in Deep Exploration (version 6.5 CSE, part of Corel DESIGNER Technical Suite X5; Corel Corporation, Ottawa, ON, Canada) for grouping, renaming, and coloring of anatomical structures. The Section Cut Tool was used to subdivide the cranial arteries. In addition, selected structures from the original 3D Embryo Atlas FBX files (including skin, neural tube, cranial nerves, and eye) were incorporated into the .u3d models.

Finally, the .u3d files were imported into Adobe Acrobat (version 2025; Adobe Inc., San Jose, CA, USA) to generate interactive 3D-PDF files containing all reconstructed structures with a custom user interface. These files can be viewed using a recent version of Adobe Acrobat Reader (freeware, Adobe Inc., San Jose, CA, USA) on Windows or macOS systems, provided that JavaScript and 3D content rendering are enabled. Interactive 3D models are provided in the Supplementary Material (Supplementary Material S1).

The 3D-PDFs were used as source images for the figures. Screenshots captured during data processing were imported into Adobe Illustrator (version 2025; Adobe Inc., San Jose, CA, USA) and arranged in multi-panel layouts. Final figures were exported for publication.

### 2.2. Literature Search Strategy

A structured literature search was performed to contextualize the embryological findings on intracranial vascular development and to support interpretation of the reconstruction data. The search was not intended as a systematic or scoping review, and the retrieved publications were not treated as primary results. The databases Embase, Medline ALL, Web of Science Core Collection, the Cochrane Central Register of Controlled Trials, and Google Scholar were searched to identify relevant literature on embryonic development of the intracranial arterial system. Database-specific search strings were developed to capture studies addressing embryological and developmental aspects of cerebral and intracranial vasculature. Search strategies are provided in the Supplementary Material (Supplementary Material S2). Retrieved records were evaluated based on predefined inclusion criteria focusing on studies describing vascular development of the brain and intracranial arteries during embryonic and early fetal stages. Selected publications were used to compare and contextualize the anatomical findings derived from the 3D reconstructions. Priority was given to classical embryological studies, foundational descriptions of human neurovascular development, and publications directly relevant to the developmental processes observed in the reconstructed specimens. Detailed information regarding the search process and study selection is provided in Supplementary Table S2.

## 3. Results

**Figure 1:**
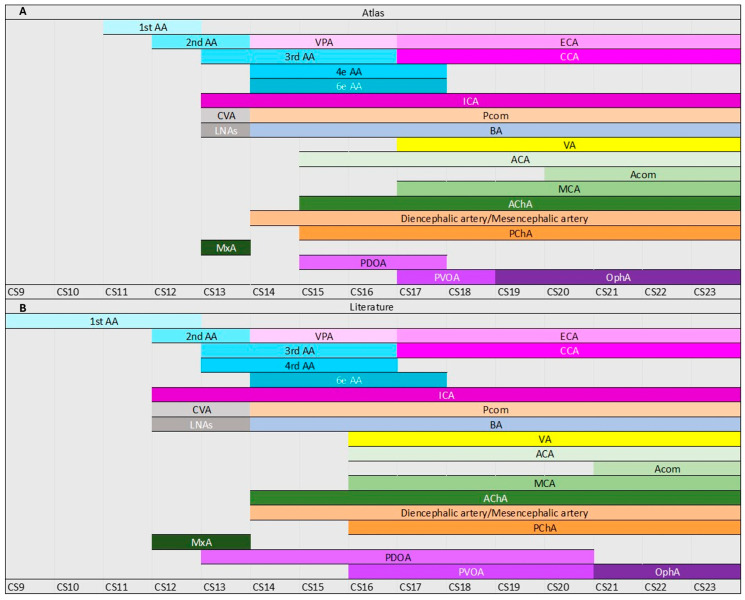
A comparison of important time points of the development of the cranial arteries during embryogenesis between our findings (**A**) compared to the analyzed literature (**B**). Colors are corresponding to arteries within Figure 2, Figure 3, Figure 4, Figure 5 and Figure 6. AA—aortic arches 1–6, VPA—ventral pharyngeal artery, ECA—external carotid artery, CCA—common carotid artery, ICA—internal carotid artery, CVA—carotid–vertebral anastomoses, Pcom—posterior communicating artery, LNAs—longitudinal neural arteries, BA—basilar artery, VA—vertebral artery, ACA—anterior cerebral artery, Acom—anterior communicating artery, MCA—middle cerebral artery, AChA—anterior choroidal artery, DiA/MeA—diencephalic/mesencephalic artery, PChA—posterior choroidal artery, MxA—maxillary artery, PDOA—primitive dorsal ophthalmic artery, PVOA—primitive ventral ophthalmic artery, OA—ophthalmic artery.

A duplicate set of thirteen embryonic stages within the Carnegie stage range from 11 to 23 were reconstructed, visualized, and subjected to analysis of neurovascular development. We undertook an in-depth examination and visualization of each stage in the vascular development of the CoW. We describe our findings hereafter according to the embryonic developmental sequence, with arterial systems described in the order of their appearance and remodeling across Carnegie stages. In the analyzed specimens, a definitive arterial configuration of the CoW is established by CS21. Subsequent stages primarily demonstrate consolidation and refinement of this configuration, with complete visual continuity consistently observed at CS23. We provide detailed insight into stage-specific developmental transitions during which anatomical variation of the CoW may arise. A comparative overview of key developmental time points in our reconstructions versus the classical literature is provided in Figure 1.

### 3.1. Aortic Arches

**Figure 2:**
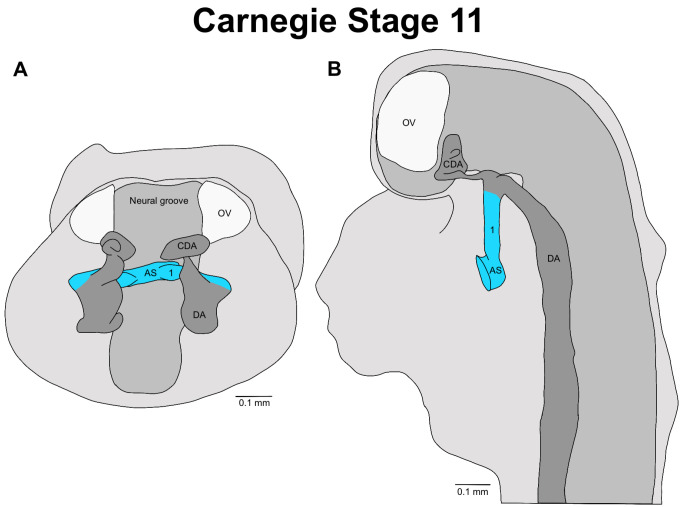
Carnegie stage 11. Cranial (**A**) and left lateral (**B**) view of the cranial arterial system in Carnegie stage 11 (CS11; specimen no. 6784; 2.5 mm; 23–26 days). CDA: cranical dorsal aorta. Light blue: aortic arch arteries. Dark gray: neural tube. All other abbreviations are defined in the manuscript’s abbreviation table. This stage illustrate the earliest recognizable cranial arterial primordia and the initial organization of the embryonic cranial circulation.

**Figure 3:**
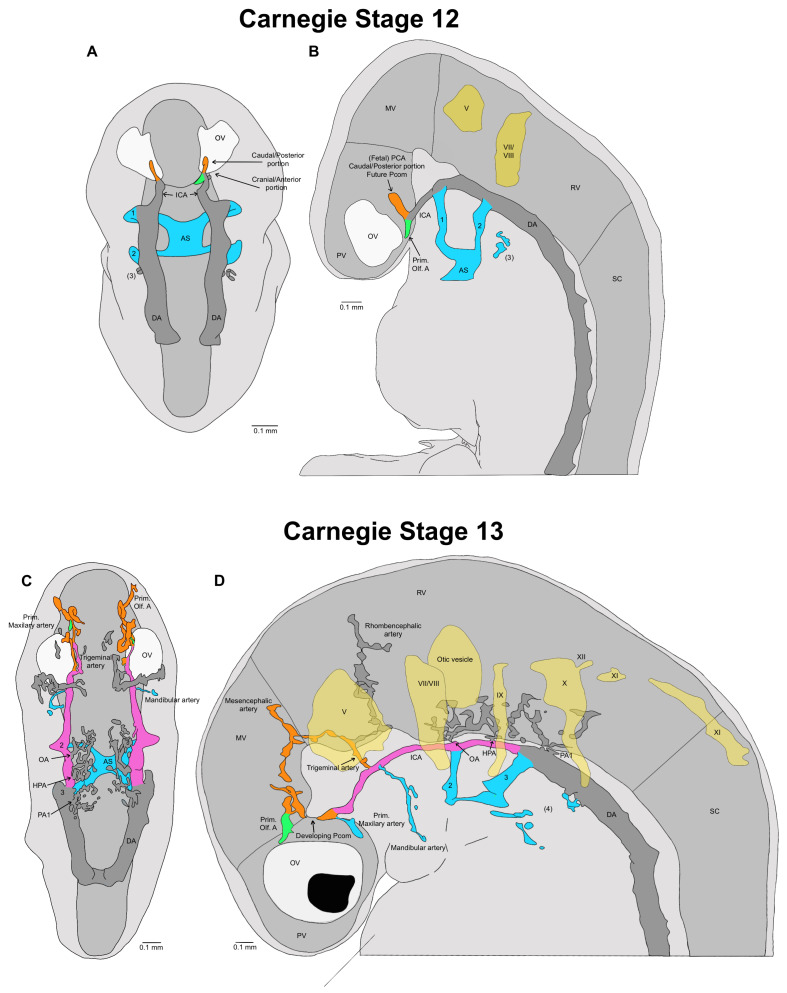
Carnegie stages 12 and 13. Cranial (**A**,**C**) and left lateral (**B**,**D**) views of the cranial arterial system in Carnegie stage 12 (CS12; specimen no. 8505A; 2.9 mm; 26–30 days) and Carnegie stage 13 (CS13; specimen no. 836; 4.1 mm; 28–32 days). Light blue: aortic arch arteries; orange: early posterior cerebral artery (future PCA); light green: primitive maxillary artery; purple: embryonic internal carotid artery. Yellow represents cranial nerves, dark gray the neural tube. All other abbreviations are defined in the manuscript’s abbreviation table. These stages are characterized by regression of the first aortic arch, persistence of the second arch derivatives, and the progressive establishment of the embryonic internal carotid artery.

The first aortic arch can be seen in the specimens at 23–26 days (CS11, 2.5 mm CRL) (Figure 2, CS11) and has completely disappeared at 28–32 days (CS13, 4.1 mm) (Figure 3, CS12, CS13). The remnant of the first aortic arch becomes the mandibular artery, which is a temporary branch of the ICA and is only visible at 28–32 days (CS13, 4.1 mm) (Figure 3, CS13). The mandibular artery is located proximal to the trigeminal artery and travels ventrally. The second aortic arch, or hyoid artery, appears at 26–30 days (CS12, 2.9 mm) and regresses at 31–35 days (CS14, 5 mm). The ventral portion of the second aortic arch is the ventral pharyngeal artery (VPA), the future external carotid artery (ECA). The third aortic arch starts to develop in the specimen at day 26–30 (CS12, 2.9 mm) but only becomes recognizable as an artery at 28–32 days (CS13, 4.1 mm). The dorsal end of the third aortic arch will become the common carotid artery (CCA) at 42–44 days (CS17, 10.6 mm) (Figure 5). Overall, the aortic arches show a tightly regulated and sequential pattern of appearance and regression, with clear temporal boundaries for the first three arches. As the aortic arches regress and remodel, their derivatives directly contribute to the formation of the early carotid arterial system.

### 3.2. Carotid Arteries

**Figure 4:**
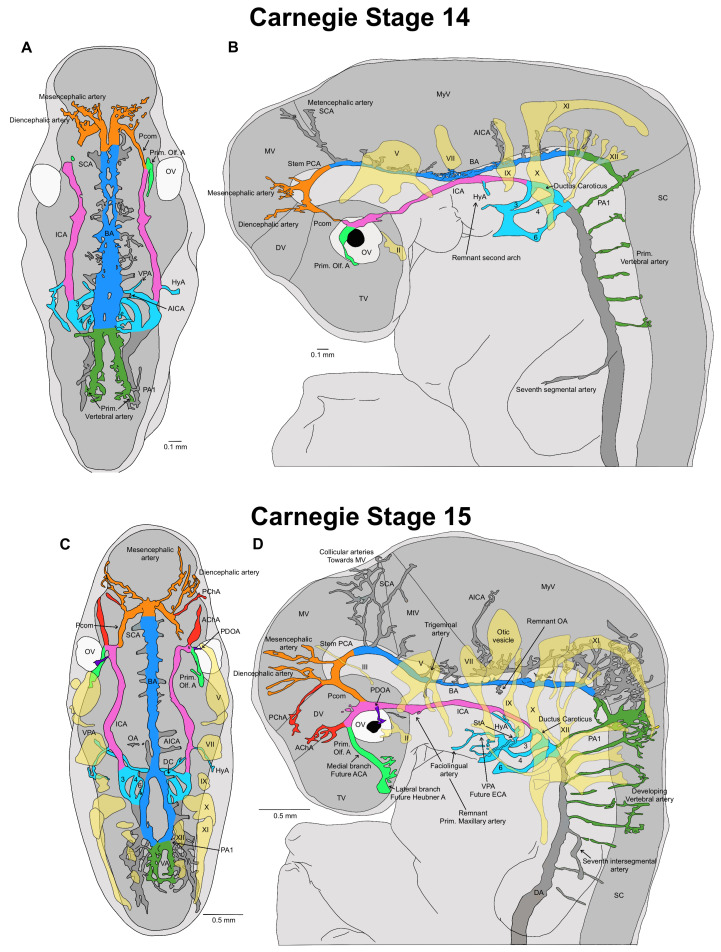
Carnegie stages 14 and 15. The cranial (**A**,**C**) and left lateral (**B**,**D**) views of the cranial arteries belonging to two Carnegie stages: CS14 (specimen no. 6502; 5.0 mm, 31–35 days) (**A**,**B**) and CS15 (specimen no. 3512; 6.6 mm, 35–38 days) (**C**,**D**). Light blue: aortic arch arteries. Dark blue: basilar artery. Orange: early posterior cerebral artery (future PCA) and branches. Light green: future stem anterior cerebral artery. Dark green: intersegmental arteries. Purple: embryonic internal carotid artery. Deep purple: primitive ventral ophthalmic artery. Red: cranial arteries. Yellow represents cranial nerves, dark gray the neural tube. All other abbreviations are defined in the manuscript’s abbreviation table. This stage is characterized by the formation of the posterior communicating artery, appearance of the transient ophthalmic arteries, and ongoing fusion of the longitudinal neural arteries.

**Figure 5:**
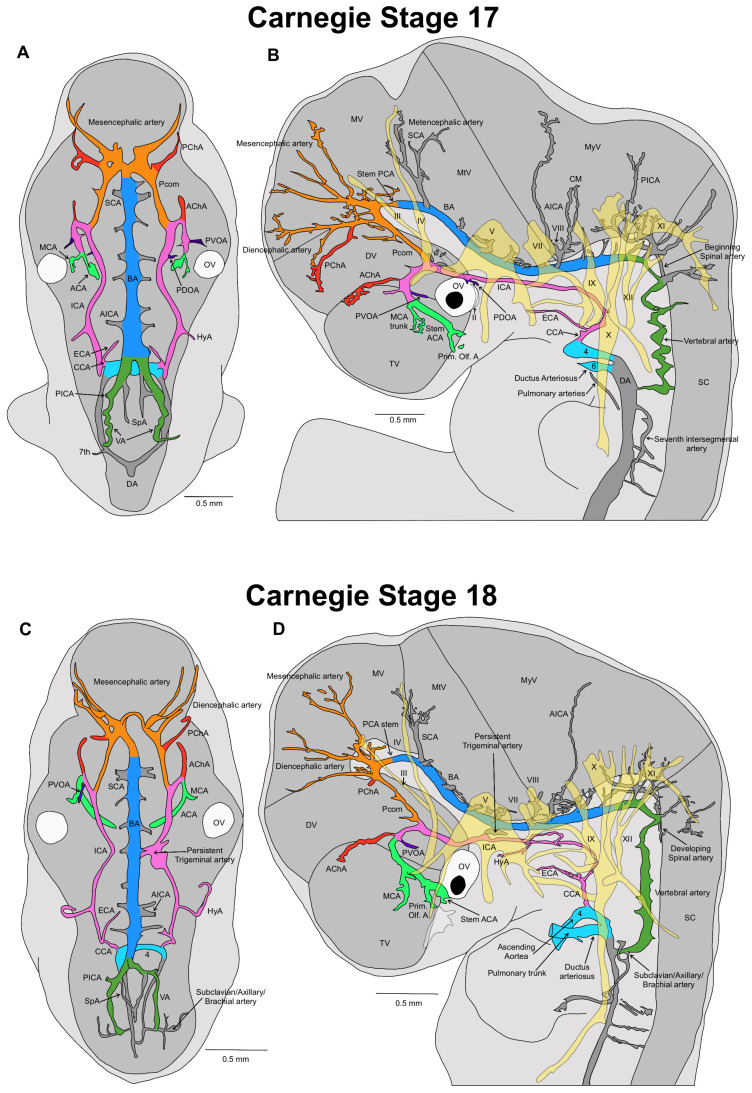
Carnegie stages 17 and 18. The cranial (**A**,**C**) and left lateral (**B**,**D**) views of the cranial arteries belonging to two Carnegie stages: CS17 (specimen no. 6521; 10.6 mm, 42–44 days) (**A**,**B**) and CS18 (specimen no. 6524; 9.7 mm, 44–48 days) (**C**,**D**). Light blue: aortic arch arteries. Dark blue: basilar artery. Orange: early posterior cerebral artery (future PCA) and branches. Light green: stem anterior cerebral artery and middle cerebral artery. Dark green: vertebral artery. Purple: common, internal, and external carotid arteries. Red: cranial arteries. Deep purple: primitive ventral ophthalmic artery. Yellow represents cranial nerves, dark gray the neural tube. All other abbreviations are defined in the manuscript’s abbreviation table. These stages are characterized by increasing differentiation of the cerebral arterial territories and continued maturation of the vertebrobasilar system. A persistent trigeminal artery remained identifiable in the analyzed CS18 specimen.

The dorsal cranial aortae already bifurcate at 26–30 days (CS12, 2.9 mm), before the embryonic ICA is even recognizable. It divides into an anterior division (cranial) and a posterior division (caudal). The embryonic ICA could be identified at 28–32 days (CS13, 4.1 mm) from the combination of both the distal portions of the dorsal aortae and the third aortic arch (Figure 3). At this stage, the involution of the first aortic arch is complete, and the second aortic arch is in the early stages of involution. Subsequently, at 31–35 days (CS14, 5.0 mm), the ICA becomes a well-defined artery as the involution of the second aortic arch is completed (Figure 4). The ICA terminates at the bifurcation of the anterior and posterior divisions. The CCA becomes recognizable at 42–44 days (CS17, 10.6 mm) when the VPA migrates caudally along the ICA. The distal portion of the VPA becomes the ECA, which further migrates in CS18 (Figure 5). Notably, the anterior and posterior divisions of the dorsal cranial aortae are already present before the embryonic ICA becomes morphologically recognizable. With the establishment of the embryonic ICA, early arterial connections between the carotid system and the developing hindbrain circulation become apparent.

### 3.3. Carotid–Vertebral Anastomoses

The embryonic ICA supplies the forebrain through the anterior division and the midbrain through its posterior division at 28–32 days (CS13, 4.1 mm) (Figure 3). The posterior division resolves into a plexus around the midbrain but does not reach the plexus at the ventral surface of the hindbrain. The vascular plexus at the hindbrain receives its supply through four presegmental arteries from the caudal ICA at 28–32 days (CS13, 4.1 mm) (Figure 3). At the level of the trigeminal ganglion, the ICA sprouts the first presegmental artery that travels dorsally to join the plexus of blood vessels at the ventral surface of the hindbrain, called the trigeminal artery. The other two presegmental arteries, called the primitive otic and hypoglossal arteries, also supply the hindbrain, but more caudally. The primitive otic artery branches off the embryonic ICA at the level of the second aortic arch and otic vesicle, and it travels with the vestibulocochlear nerve (VIII) to the vascular plexus at the hindbrain. The hypoglossal artery is located behind the otic artery in the caudal direction and travels along the glossopharyngeal nerve (IX) to the vascular plexus. Finally, we identified the proatlantal artery (type 1), a cervical intersegmental artery supplying the hindbrain just below the cranial extension of the embryonic ICA (42–44 days, CS17, 10.6 mm) (Figure 5). Together, these presegmental arteries provide the sole arterial supply to the hindbrain prior to the establishment of a permanent carotid–basilar connection. While these carotid–vertebral connections provide the initial arterial supply to the hindbrain, additional branches of the embryonic ICA simultaneously contribute to vascularization of the forebrain and optic region.

### 3.4. Ophthalmic Artery

At 28–32 days (CS13, 4.1 mm), another branch of the primitive embryonic ICA can be observed, namely the primitive maxillary artery. This artery is the next branch after the mandibular artery in the cerebral continuation of the ICA. It extends cranially along the ventral wall of the forebrain and supplies the base of the optic vesicle (Figure 3).

**Figure 6:**
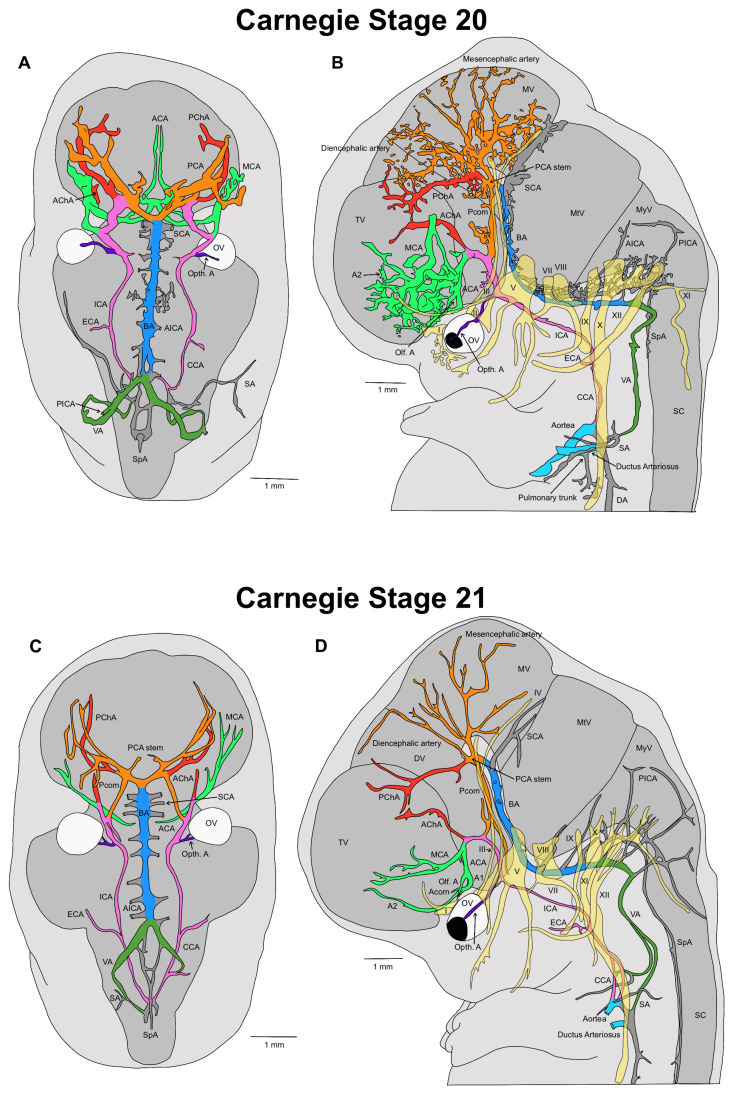
Carnegie stages 20 and 21. The cranial (**A**,**C**) and left lateral (**B**,**D**) views of the cranial arteries belonging to two Carnegie stages; CS20 (specimen no. 462; 15.9 mm, 51–53 days) (**A**,**B**) and CS21 (specimen no. 7254; 17.3 mm, 53–54 days) (**C**,**D**). Light blue: remnants of aortic arch arteries. Dark blue: basilar artery. Orange: posterior cerebral artery and branches. Light green: stem anterior cerebral artery and middle cerebral artery. Bright yellow: vertebral artery. Purple: common, internal, and external carotid arteries. Deep purple: ophthalmic artery. Red: cranial arteries. Yellow represents cranial nerves, dark gray the neural tube. All other abbreviations are defined in the manuscript’s abbreviation table. These stages represent completion of the embryonic configuration of the CoW, with anterior and posterior communicating arteries established and vertebrobasilar connections fully developed.

At 35–38 days (CS15, 6.6 mm), the primitive maxillary artery almost completely regresses and the primitive dorsal ophthalmic artery (PDOA) takes over the supply to the optic vesicle (Figure 4). The PDOA originates from the embryonic ICA proximal to the terminal bifurcation and finally courses toward the lens contributing to the early hyaloid vascular system supplying the optic vesicle. At 42–44 days (CS17, 10.6 mm), the primitive ventral ophthalmic artery (PVOA), arises from the anterior division of the ICA at the level of AChA. Both arteries are responsible for the supply of blood to the optic vesicle at this stage (Figure 5). In the analyzed CS18 specimens (44–48 days, 9.7 mm), the PDOA had regressed, leaving the PVOA as the sole arterial supply to the optic vesicle until CS20 (51–53 days, 15.9 mm). At this stage, the definitive ophthalmic artery (OA) arises from the ICA at the level of the clinoidal segment, as a continuation of the PVOA. (Figure 6). Thus, the blood supply to the optic vesicle is characterized by a sequence of transient arterial sources before the establishment of the definitive OA. In parallel with the evolving ophthalmic circulation, further differentiation of the anterior and posterior divisions of the ICA defines the emerging cerebral arterial territories.

### 3.5. Posterior Divisions of the ICA

The posterior division of the ICA will become responsible for the supply to the occipital lobe and the cerebellum, which starts at 28–32 days (CS13, 4.1 mm) when it courses toward the developing BA (Figure 3). A permanent connection between the posterior division and the developing BA was observed at 31–35 days (CS14, 5.0 mm). This connecting artery is the posterior communicating artery (Pcom) which takes over the supply to the posterior circulation from the trigeminal artery (Figure 4). Also, two branches are already extending off from the caudal portion of the Pcom: one supplies the mesencephalon (the mesencephalic artery) and the other the diencephalon (the diencephalic artery) (Figure 4). At this stage, the posterior circulation remains predominantly carotid-dependent despite the emerging basilar system.

### 3.6. Anterior Divisions of the ICA

The primitive olfactory artery originating from the ICA extends in a medial direction around the forebrain and the optic vesicle to the olfactory area at 31–35 days (CS14, 5.0 mm) (Figure 4). At 35–38 days (CS15, 6.6 mm), the primitive olfactory artery courses toward the nasal fossa and and gives rise to the stem of the anterior cerebral artery (ACA), which sends offshoots to the contralateral ACA (Figure 4). As development progresses, the primitive olfactory artery involutes while the ACA enlarges, ultimately reducing the primitive olfactory artery to a small branch of the ACA. A vascular plexus can be observed at the ventral surface of the telencephalon at 44–48 days (CS18, 9.7 mm) (Figure 5), whereas by CS20 (51–53 days, 15.9 mm) an anterior communicating artery connecting the bilateral ACAs was observed (Figure 6). This anterior communicating artery (Acom) completes the CoW. At 51–53 days (CS20, 15.9 mm), both ACAs have extended cranially between the two hemispheres (Figure 6). This marks the transition from a plexiform anterior circulation to a defined arterial configuration. The middle cerebral artery (MCA) is first identified as a small vessel originating from the stem of the ACA at 42–44 days (CS17, 10.6) (Figure 5). Later, at 44–48 days (CS18, 9.7 mm), the MCA becomes more prominent (Figure 5). The MCA develops fast and is already responsible for the supply of a large cerebral cortical territory at 51–53 days (CS20, 15.9 mm) (Figure 6). The MCA shows rapid enlargement over a short developmental interval, quickly becoming a dominant supplier of the lateral cerebral cortex. As the major cerebral arteries expand, deep cerebral structures are supplied through developing anterior and posterior choroidal arteries.

### 3.7. Choroidal Arteries

The anterior choroidal artery (AChA) was observed arising from the proximal portion of the anterior division of the ICA at 35–38 days (CS15, 6.6 mm) (Figure 4). The AChA lies distal to the Pcom and courses cranially through the groove between the telencephalon and the diencephalon, where it divides into several branches. During the embryonic period, mostly the choroidal stage, the AChA supplies significant regions of the telencephalon. At 35–38 days (CS15, 6.6 mm), another branch extends from the stem of the diencephalic artery, which presents the posterior choroidal artery (PChA) (Figure 4). The PChA branches upon the lateral wall of the diencephalon, and eventually anastomoses with branches of the AChA at 51–53 days (CS20, 15.9 mm) (Figure 6). During the embryonic period, the telencephalon is supplied by parallel anterior and posterior choroidal systems that later establish interconnections. Concurrent with maturation of the carotid-derived cerebral arteries, the posterior circulation develops through fusion of the longitudinal neural arteries (LNAs) and formation of the vertebrobasilar system.

### 3.8. Vertebral and Basilar Artery

At 28–32 days (CS13, 4.1 mm), the emergence of the posterior circulation can be observed as two separate islands consisting of a plexus of blood vessels (Figure 3). Each island is located along the midline of the hindbrain and is the predecessor of the LNAs. However, at 31–35 days (CS14, 5.0 mm), the LNAs are already partially fused across the midline of the hindbrain at two points to form the BA, one in the region of the anterior inferior cerebellar artery (AICA) and the other more rostrally in the region of the superior cerebellar artery (SCA). This pattern suggests that BA formation involves fusion events occurring at multiple sites along the LNAs rather than a strictly craniocaudal progression. At this stage, a plexiform segment is recognizable at the midline of BA, and the cranial tip of the BA is unfused. In the analyzed CS15 specimens (35–38 days, 6.6 mm) fusion of the LNAs was observed, resulting in the formation of the BA (Figure 4).

The transverse longitudinal anastomoses between the first six cervical intersegmental arteries begin to develop at 35–38 days (CS15, 6.6 mm), recognizable as a plexus extending from the proatlantal artery caudally toward the sixth intersegmental artery (Figure 4). The intersegmental arteries subsequently become longitudinally connected, forming the vertebral artery (VA) at 42–44 days (CS17, 10.6 mm) (Figure 5). At this point, the VA loses most of its connection with the dorsal aortae (DA) at 42–44 days (CS17, 10.6 mm) through involution of the cervical intersegmental arteries (Figure 5). The cranial ends of the developing vertebral arteries subsequently establish connections with the caudal LNAs, thereby linking the vertebral system to the forming BA. The seventh cervical intersegmental artery persisted in the analyzed specimens and is consistent with the future definitive origin of the VA from the subclavian artery at 44–48 days (CS18, 9.7 mm) (Figure 5). At these stages, fusion of the LNAs remains incomplete and is characterized by segmental connections and midline fenestration. Together, these arterial systems establish the framework of the mature intracranial circulation by the end of the embryonic period.

## 4. Discussion

The aim of this study is to provide an updated embryological framework of neurovascular development, with particular focus on the formation of the CoW, to improve understanding of stage-specific anatomical variation. By comparing our reconstructions with the existing literature, several refinements in developmental timing and vascular configuration can be identified.

### 4.1. Pharyngeal Arch Arteries Development

Current knowledge of the development of the human pharyngeal arch arteries is largely based on the classical work of Congdon (1922) and later complemented by Rana et al. (2014). Congdon reported that the first aortic arch artery can already be seen in human embryos of approximately 1.3 mm (CS8) and regresses as the fourth aortic arch appears [11,24,25].

In our series, however, the first aortic arch artery could be identified at CS11 and remained observable until the end of CS13, followed by regression shortly before the third arch became recognizable. These observations suggest a slightly different developmental timing than originally proposed by Congdon. Similarly, our observations indicate that the fourth and sixth arches appear simultaneously. These differences may partly reflect methodological constraints in early descriptions and are supported by more recent 3D reconstructions of pharyngeal arch development, which emphasize interspecies variability [14].

### 4.2. Intracranial Arteries and the Trigeminal Artery

Padget (1948) provided a detailed description of the development of the intracranial arteries that is still widely cited. She described the primitive trigeminal artery as a branch of the first aortic arch that travels dorsally with the trigeminal ganglion to the primordial hindbrain channel [22,26]. This corresponds to CS11–12. In our specimens, no artery branching from the first arch could be identified at these stages. Instead, we observed a cranial continuation of the dorsal aorta extending beyond the first aortic arch toward the superficial vascular plexus surrounding the optic vesicle. In the analyzed specimens, the definitive trigeminal artery was first observed at CS13, arising from the embryonic (ICA) and terminating in the developing (LNAs). This aligns with Padget’s “second stage” of trigeminal artery development but suggests a later timing. Interestingly, a persistent trigeminal artery remained identifiable in the analyzed CS18 specimen (Figure 5). This observation suggests that regression of embryonic carotid–vertebrobasilar connections may extend into later stages of intracranial arterial development and provides a developmental framework for understanding persistent carotid–vertebrobasilar connections encountered in the mature cerebrovascular system.

Padget also described BA formation as a craniocaudal fusion of the two LNAs between CS12 and CS15 [26]. In contrast, our reconstructions demonstrate a more complex overlapping process. We observed the LNAs to first appear as bilateral vascular islands along the rhombencephalon, which subsequently coalesce into longitudinal arteries. Our observations support a model in which fusion into the BA occurs simultaneously in both craniocaudal and caudocranial directions.

### 4.3. Role of the Notochord in Fusion of the LNAs

Our observations suggest that fusion of LNAs to form the BA may be influenced by the spatial relationship between the notochord and neural tube, facilitating midline vascular fusion. A similar mechanism has been proposed for the fusion of the paired dorsal aortae [27,28]. Incomplete or asymmetric fusion of the LNA may result in segmental non-fusion of the BA, which in adult anatomy is commonly described as BA fenestration. Such unfused segments are frequently located in the caudal BA and may represent predilection sites for aneurysm formation [29,30,31]. We hypothesize that insufficient caudal retraction of the notochord could contribute to incomplete fusion. This mechanism parallels other midline fusion defects, such as neural tube closure failure leading to spina bifida [32]. Notably, duplications of the pituitary gland, which also arise under notochordal influence, have been associated with vascular fenestrations [33,34], further supporting this developmental link. The developmental sequence observed between CS13 and CS15 further illustrates how variation in the fusion process of the paired LNAs may influence the final morphology of the BA. Persistence of localized non-fused segments may provide an embryological framework for understanding BA fenestrations encountered in adult cerebrovascular anatomy.

### 4.4. Ophthalmic Artery Development

The OA remains one of the most debated vessels in neuroembryology due to its complex ontogeny. Multiple embryological systems contribute to its development, including the embryonic ICA, the stapedial artery, and the aortic arches [35,36,37,38].

Padget (1948) described six developmental stages, beginning with the mandibular and maxillary arteries, followed by the PDOA and PVOA. In her scheme, the definitive OA arises from the PDOA, while the PVOA regresses. In our material, however, both PDOA and PVOA appeared later than in Padget’s description. More importantly, our reconstructions suggest that the PDOA regresses before the PVOA and support the interpretation that the definitive OA may arise from the persisting PVOA.

This interpretation is consistent with the model of Lasjaunias (2001), who, based on angiographic variants, proposed that the proximal PVOA regresses into the definitive OA. Our observations from 3D reconstructions are more consistent with Lasjaunias’ than with Padget’s and provide anatomical support for this interpretation [38]. The coexistence and subsequent remodeling of multiple primitive ophthalmic arterial channels observed between CS16 and CS20 further illustrate how variation in persistence or regression of these vessels may influence the final OA configuration encountered in adult anatomy.

### 4.5. Timing and Variability

Some discrepancies were also observed in the timing of cranial artery development when compared to earlier research (see Figure 1). For example, the emergence of the PDOA and PVOA occurred later than described by Padget (1948), while the appearance of the aortic arches did not fully match Congdon’s (1922) timeline. Such differences may result from variability in specimen quality, fixation, or histological interpretation, highlighting the value of serial 3D reconstructions for re-evaluating established timelines. Our openly available dataset, although limited in sample size, provides unique serial reconstructions that allow for re-evaluation of these developmental sequences and should be interpreted as a stage-based developmental framework rather than an exact chronological timeline applicable to all embryos (3Dembryoatlas.com).

### 4.6. Clinical Relevance

Variations of the CoW and its contributing arteries are frequently encountered in clinical practice, particularly in the context of stroke, aneurysm formation, and neurosurgical planning. From a clinical perspective, the configuration of the CoW influences the capacity for collateral circulation during arterial occlusion, which may affect stroke severity and outcome. Several sites of intracranial aneurysm formation correspond to regions of complex vascular remodeling during embryogenesis, such as the Acom artery complex and the BA apex. Our findings provide an embryological framework that places commonly encountered vascular configurations of the CoW within their developmental context, emphasizing the role of timing and vascular remodeling during defined Carnegie stages. The stage-resolved reconstructions further demonstrate the dynamic transition from a predominantly carotid-dependent posterior circulation toward a vertebrobasilar-dominant configuration. Variation in this remodeling process may contribute to differences in posterior cerebral arterial anatomy encountered in the mature cerebrovascular system. Recent clinical studies have further demonstrated that anatomical variants of the CoW influence collateral capacity during carotid artery stenosis and carotid endarterectomy, thereby affecting perioperative neurological risk and cerebral ischemic outcome. Furthermore, a comprehensive review by Jones et al. (2021) emphasizes the importance of understanding developmental variation of the CoW for the interpretation of cerebrovascular anatomy and neurovascular interventions [39,40,41].

### 4.7. Limitations

In total, 26 human embryos were imaged and analyzed covering the complete developmental window of the CoW. Although analyzing only two specimens per stage may not fully capture developmental variance, the inclusion of all relevant stages allows for the identification of consistent growth patterns. It should also be noted that all reconstructions relied on manual vascular annotations. Despite meticulous attention to detail, minor structures may have been overlooked or misinterpreted. In addition, *Z*-axis resolution varied between embryos, which meant that vascular structures were easier to trace in some specimens than in others, potentially affecting the visibility of very small or transient vessels. Furthermore, differences in specimen preservation quality and occasional histological sectioning artifacts inherent to historical embryological collections may also have influenced visualization of small vascular structures.

## 5. Conclusions

This study refines the classical description of intracranial arterial development from a three-dimensional perspective. Our stage-resolved reconstructions support a model of bidirectional fusion during basilar artery formation and indicate that the definitive ophthalmic artery predominantly derives from the primitive ventral ophthalmic artery rather than the primitive dorsal ophthalmic artery. In the analyzed specimens, the definitive arterial configuration of the circle of Willis appeared largely established by Carnegie stage 21, after which no new arterial connections were observed. Subsequent stages (Carnegie stages 22–23) predominantly reflect morphological consolidation and refinement, with complete visual continuity of the circle of Willis consistently observed at Carnegie stage 23. Anatomical variants may originate from disruption or persistence of developmental processes occurring within this critical developmental window. Together, these insights bridge embryology and clinical neurovascular disease by providing a developmental framework for interpreting vascular anatomical variation encountered in stroke, cerebrovascular anomalies and neurosurgical practice.

## Data Availability

The interactive 3D reconstructions generated during this study will be made publicly available through the 3D Human Development platform (https://www.3dhumandevelopment.com/3d-neurovascular-atlas/ (accessed on 7 May 2026)). This resource forms part of a broader collection of digital developmental atlases and educational tools. Furthermore are the interactive 3D-PDF models associated with this study provided as Supplementary Material. Additional data supporting the findings of this study are available from the corresponding author upon reasonable request.

## Supplementary Materials

The following supporting information can be downloaded at: https://www.mdpi.com/article/10.3390/life16071153/s1. Supplementary Material S1: Interactive 3D-PDF Models of Human Embryonic Intracranial Arterial Development (Carnegie Stages 11–23); Supplementary Material S2: Database Search Strategy; Table S1: Overview of the specimens used for the reconstruction; Table S2: Literature search strategy and study

## Abbreviations

The following abbreviations are used in this manuscript:

**Abbreviation**	**Definition**
AA	Aortic Arches
ACA	Anterior Cerebral Artery
AChA	Anterior Choroidal Artery
Acom	Anterior Communicating Artery
AICA	Anterior Inferior Cerebellar Artery
AS	Aortic Sac
BA	Basilar Artery
CCA	Common Carotid Artery
CD	Carotid Duct
CoW	Circle of Willis
CRL	Crown–Rump Length
CS	Carnegie Stage
CVA	Carotid–Vertebral Anastomosis
DA	Dorsal Aortae
DiA	Diencephalic Artery
DV	Diencephalic Vesicle/Diencephalon
ECA	External Carotid Artery
HA	Heubner Artery (Distal medial striate artery)
HpA	Hypoglossal Artery
HyA	Hyoid Artery
ICA	Internal Carotid Artery
LNA	Longitudinal Neural Artery
MCA	Middle Cerebral Artery
MeA	Mesencephalic Artery
MtV	Metencephalic Vesicle/Metencephalon
MV	Mesencephalic Vesicle/Mesencephalon
MxA	Maxillary Artery
MyV	Myelencephalic Vesicle/Myelencephalon
OA	Ophthalmic Artery
OlfA	Olfactory Artery
OpticA	Optic Artery
OV	Otic Vesicle
PA	Proatlantal Artery (type 1/type 2)
PCA	Posterior Cerebral Artery
PChA	Posterior Choroidal Artery
Pcom	Posterior Communicating Artery
PDOA	Primitive Dorsal Ophthalmic Artery
PICA	Posterior Inferior Cerebellar Artery
PV	Prosencephalic Vesicle/Prosencephalon
PVOA	Primitive Ventral Ophthalmic Artery
RP	Rathke’s Pouch
RV	Rhombencephalic Vesicle/Rhombencephalon
SC	Spinal Cord
SCA	Superior Cerebellar Artery
SpA	Spinal Artery
StA	Stapedial Artery
TrA	Trigeminal Artery
TV	Telencephalic Vesicle/Telencephalon
VA	Vertebral Artery
VPA	Ventral Pharyngeal Artery
I	Olfactory Nerve
II	Optic Nerve
III	Oculomotor Nerve
IV	Trochlear Nerve
V	Trigeminal Nerve
VI	Abducens Nerve
VII	Facial Nerve
VIII	Vestibulocochlear Nerve
IX	Glossopharyngeal Nerve
X	Vagus Nerve
XI	Accessory Nerve
XII	Hypoglossal Nerve
