# Supplementary material for: Vascular Neuroembryology: The Genesis of the Intracranial Arterial System and the Circle of Willis
Source: Life (Basel). 2026 Jul 13;16(7):1153. doi: 10.3390/life16071153 (PMC13412907; doi:10.3390/life16071153)
Supplement: Supplementary file 1 [file life-16-01153-s001.zip › Supplementary Material S2.pdf]

life

MDPI

Supplementary Material S2: Database Search Strategy

Embase 1414

('brain blood vessel'/de OR 'brain artery'/exp OR ('blood vessel'/de AND ('brain'/de OR 'brain development'/exp)) OR (((brain* OR cerebr* OR communicating OR basilar* OR meningeal* OR pia OR pial OR cerebel* OR intracran*) NEAR/6 (arter* OR vessel* OR vascular-system* OR vasculature*)) OR ((vascular) NEAR/3 (bed)) OR ((basilar*) NEAR/3 (circulation*)) OR ((willis*) NEAR/3 (circ* OR polygon*))):ab,ti,kw) AND ('prenatal development'/exp OR 'brain development'/exp OR (((embry* OR foet* OR fetal* OR fetus* OR prenatal* OR intrauterin*) NEAR/9 (development* OR developing OR evolution OR morphogenes* OR maturation* OR viabilit*))):ab,ti,kw) NOT ((animal/exp OR animal*:de OR nonhuman/de) NOT ('human'/exp))

Medline 1624

(((exp Cerebral Arteries/ OR Basilar Artery/ OR Meningeal Arteries/ OR (Arteries/ AND Brain/) OR (((brain* OR cerebr* OR communicating OR basilar* OR meningeal* OR pia OR pial OR cerebel* OR intracran*) ADJ6 (arter* OR vessel* OR vascular-system* OR vasculature*)) OR ((vascular) ADJ3 (bed)) OR ((basilar*) ADJ3 (circulation*)) OR ((willis*) ADJ3 (circ* OR polygon*))).ab,ti,kw.) AND (exp Embryonic Development/ OR exp Fetal Development/ OR (((embry* OR foet* OR fetal* OR fetus* OR prenatal* OR intrauterin*) ADJ9 (development* OR developing OR evolution OR morphogenes* OR maturation* OR viabilit*))).ab,ti,kw.)) OR (exp Cerebral Arteries/em OR exp Cerebral Arteries/gd OR Basilar Artery/em OR Meningeal Arteries/em OR Basilar Artery/gd OR Meningeal Arteries/gd)) NOT (exp animals/ NOT humans/)

Web of Science 478

TS=(((((brain* OR cerebr* OR communicating OR basilar* OR meningeal* OR pia OR pial OR cerebel* OR intracran*) NEAR/5 (arter* OR vessel* OR vascular-system* OR vasculature*)) OR ((vascular) NEAR/2 (bed)) OR ((basilar*) NEAR/2 (circulation*)) OR ((willis*) NEAR/2 (circ* OR polygon*)))) AND ((((embry* OR foet* OR fetal* OR fetus* OR prenatal* OR intrauterin*) NEAR/9 (development* OR developing OR evolution OR morphogenes* OR maturation* OR viabilit*)))) NOT ((animal* OR rat OR rats OR mouse OR mice OR murine OR dog OR dogs OR canine OR cat OR cats OR feline OR rabbit OR cow OR cows OR bovine OR rodent* OR sheep OR ovine OR pig OR swine OR porcine OR veterinar* OR chick* OR zebrafish* OR baboon* OR nonhuman* OR primate* OR cattle* OR goose OR geese OR duck OR macaque* OR avian* OR bird* OR fish*) NOT (human* OR patient* OR women OR woman OR men OR man)))

Cochrane 8

((((brain* OR cerebr* OR communicating OR basilar* OR meningeal* OR pia OR pial OR cerebel* OR intracran*) NEAR/6 (arter* OR vessel* OR vascular-system* OR vasculature*)) OR ((vascular) NEAR/3 (bed)) OR ((basilar*) NEAR/3 (circulation*)) OR ((willis*) NEAR/3 (circ* OR polygon*))):ab,ti,kw) AND ((((embry* OR foet* OR fetal* OR fetus* OR prenatal* OR intrauterin*) NEAR/9 (development* OR developing OR evolution OR morphogenes* OR maturation* OR viabilit*))):ab,ti,kw)

Google Scholar 200

'brain|cerebral|intracranial artery|arteries|arterial|vessel|willis' 'embryo|foetus|fetal|fetus|prenatal|intrauterine development|developing|developed|maturation' -mouse -rat -animal

Table S1. Overview of the specimens used for the reconstruction

CS	Specimen #	Origin	Year	Acquired through	CRL (mm)	Days post fertilization
11	6784 *	CC	1933	Hysterectomy	2.46	23-26
11	6344	CC	1931	Hysterectomy	2.58	
12	8505A *	CC	1947	Miscarriage	2.86	26-30
12	8943	CC	1934	Hysterectomy	3.58	
13	5541	CC	1927	Miscarriage	4.08	28-32
13	836 *	CC	1914	Hysterectomy	4.09	
14	8314	CC	1945	Hysterectomy	5.16	31-35
14	6502 *	CC	1931	No information	5.54	
15	721	CC	1913	No information	4.79	35-38
15	3512 *	CC	1921	Miscarriage	6.55	
16	8773	CC	1950	Therapeutic abortion	6.74	37-42
16	6517	CC	1931	No information	10.46	
17	6521 *	CC	1933	No information	10.60	42-44
17	6520	CC	1932	No information	12.21	
18	6524 *	CC	1933	No information	9.73	44-48
18	4430	CC	1923	No information	15.85	
19	2114	CC	1918	Hysterectomy	12.59	45-51
19	8965	CC	1952	Abortion (EUG)	17.72	
20	462 *	CC	1910	Miscarriage	15.93	51-53
20	s2025	AUMC	~1975	No information	19.77	
21	7254 *	CC	1936	Hysterectomy	17.36	53-54
21	4090	CC	1922	Abortion (EUG)	19.43	
22	895	CC	1914	Hysterectomy	21.22	54-58
22	H983	BC	1962	No information	28.00	
23	950	CC	1914	Miscarriage	23.79	56-60
23	9226	CC	1954	Abortion (EUG)	30.01	

CS: Carnegie stage, Specimen #: * used for vascular 3D-atlas, Year: Year of acquisition, CRL: Calculated crown-rump-length in mm, Days: Days of development; CC: Carnegie Collection, BC: Boyd Collection, AUMC = Amsterdam University Medical Center.

Supplementary Table S2. Literature search strategy and study

Database searched	Via	Years of coverage	Records	Records after duplicates removed
**Embase**	Embase.com	1971 - Present	1414	1397
**Medline ALL**	Ovid	1946 - Present	1624	1048
**Web of Science Core Collection**	Web of Knowledge	1975 - Present	478	151
**Cochrane Central Register of Controlled Trials**	Wiley	1992 - Present	8	2
**Other sources: Google Scholar (200 top-ranked)**			200	158
**Total**			**3724**	**2756**
**After screening on title/abstract and full text**			**Total 42 included**	

Disclaimer/Publisher's Note: The statements, opinions and data contained in all publications are solely those of the individual author(s) and contributor(s) and not of MDPI and/or the editor(s). MDPI and/or the editor(s) disclaim responsibility for any injury to people or property resulting from any ideas, methods, instructions or products referred to in the content.

https://doi.org/10.3390/life16071153
