# Supplementary material for: Vascular Neuroembryology: The Genesis of the Intracranial Arterial System and the Circle of Willis
Source: Life (Basel). 2026 Jul 13;16(7):1153. doi: 10.3390/life16071153 (PMC13412907; doi:10.3390/life16071153)

# Carnegie stage 21 (53-54 days, 17.3 mm) specimen 7254

Amsterdam UMC ACTA Academic Centre for Dentistry Amsterdam

All structures

Skin

Neural tube

Cranial nerves

Eye

Arteries all

Arteries left (L)

Arteries right (R)

Telenchepalic arteries

Choroidal arteries

Di/Mes-encephalic arteries

Basilar artery

Met/Myl-encephalic arteries

Vertebral arteries and branches

Carotid arteries

Orbital arteries

Pharyngeal arch derivatives

Aorta and branches

Selected structure:

Use Adobe Reader with preferred settings (see below)

< Click here to enable 3D interaction >

**Technical Note**

View this PDF file in a recent version of Adobe Acrobat Reader: https://get.adobe.com/reader/

3D interaction is only possible on MS Windows or Mac OS. Javascript and playing of 3D content must be enabled by making these settings in Adobe Reader; *Menu*, *Preferences*:

1) In *JavaScript*
   - enable *Enable Acrobat JavaScript*
2) In *Multimedia & 3D*
   - enable *Enable playing of 3D content*
   - disable *Show 3D Orientation Axis*
   - *Optimization Scheme for Low Framerate: None*

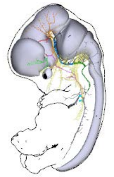

Overview

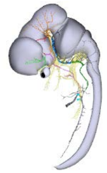

Neurovascular

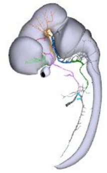

Vascular + Neural tube

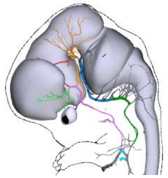

Zoom overview

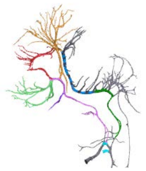

Lateral view arterial system

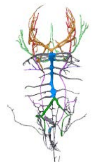

Cranial view arterial system

# Carnegie stage 21 (53-54 days, 17.3 mm) specimen 7254

Amsterdam UMC

ACTA Academic Centre for Dentistry Amsterdam

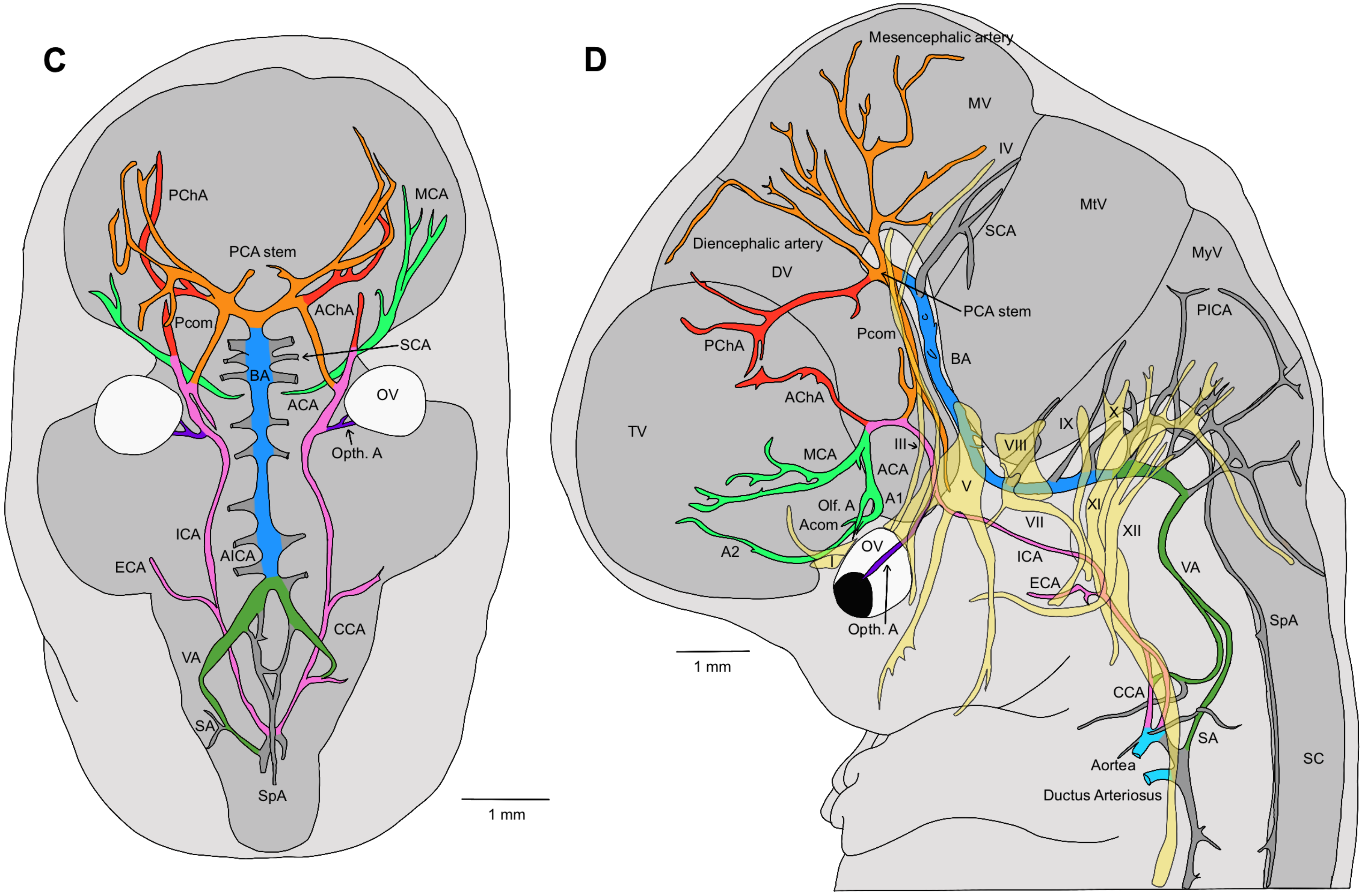

Carnegie stage 21 (53-54 days, 17.3 mm) specimen 7254

Amsterdam UMC

ACTA Academic Centre for Dentistry Amsterdam

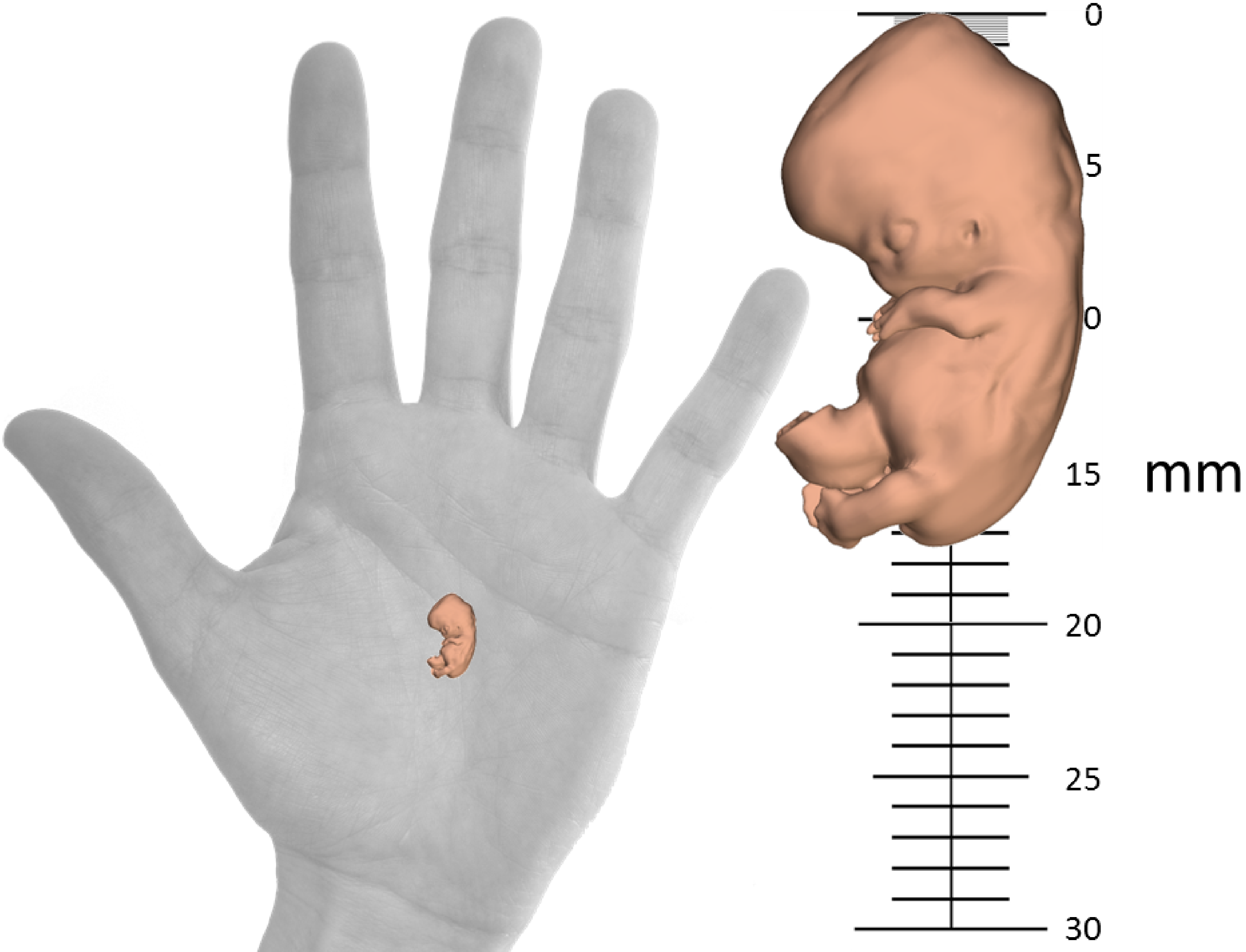

Supplement: Supplementary file 1 [file life-16-01153-s001.zip › Supplementary Material S1-Interactive 3D-PDF Models of Human Embryonic Intracranial Arterial Development (Carnegie Stages 11–23) .zip!/3DAtlas_CS21-7254_NV_v1_3D.pdf]
