# Supplementary material for: Vascular Neuroembryology: The Genesis of the Intracranial Arterial System and the Circle of Willis
Source: Life (Basel). 2026 Jul 13;16(7):1153. doi: 10.3390/life16071153 (PMC13412907; doi:10.3390/life16071153)

Carnegie stage 12 (26-30 days, 2.9 mm) specimen 8505A

All structures
Skin
Neural tube
Cranial nerves
Eye
Arteries all
Arteries left
Arteries right
Primitive olfactory arteries
Posterior communicating arteries
Cranial dorsal aorta
Pharyngeal arch arteries
Aorta and branches

Selected structure:

Use Adobe Reader with preferred settings (see below)

< Click here to enable 3D interaction >

Technical Note

View this PDF file in a recent version of Adobe Acrobat Reader: https://get.adobe.com/reader/

3D interaction is only possible on MS Windows or Mac OS. Javascript and playing of 3D content must be enabled by making these settings in Adobe Reader; *Menu, Preferences*:

1) In *JavaScript*
   - enable *Enable Acrobat JavaScript*
2) In *Multimedia & 3D*
   - enable *Enable playing of 3D content*
   - disable *Show 3D Orientation Axis*
   - *Optimization Scheme for Low Framerate: None*

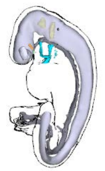
Overview

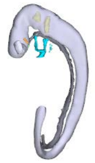
Neurovascular

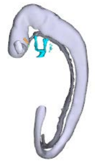
Vascular + Neural tube

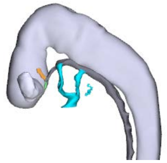
Zoom overview

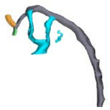
Lateral view arterial system

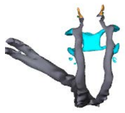
Cranial view arterial system

Carnegie stage 12 (26-30 days, 2.9 mm) specimen 8505A

Amsterdam UMC

ACTA Academic Centre for Dentistry Amsterdam

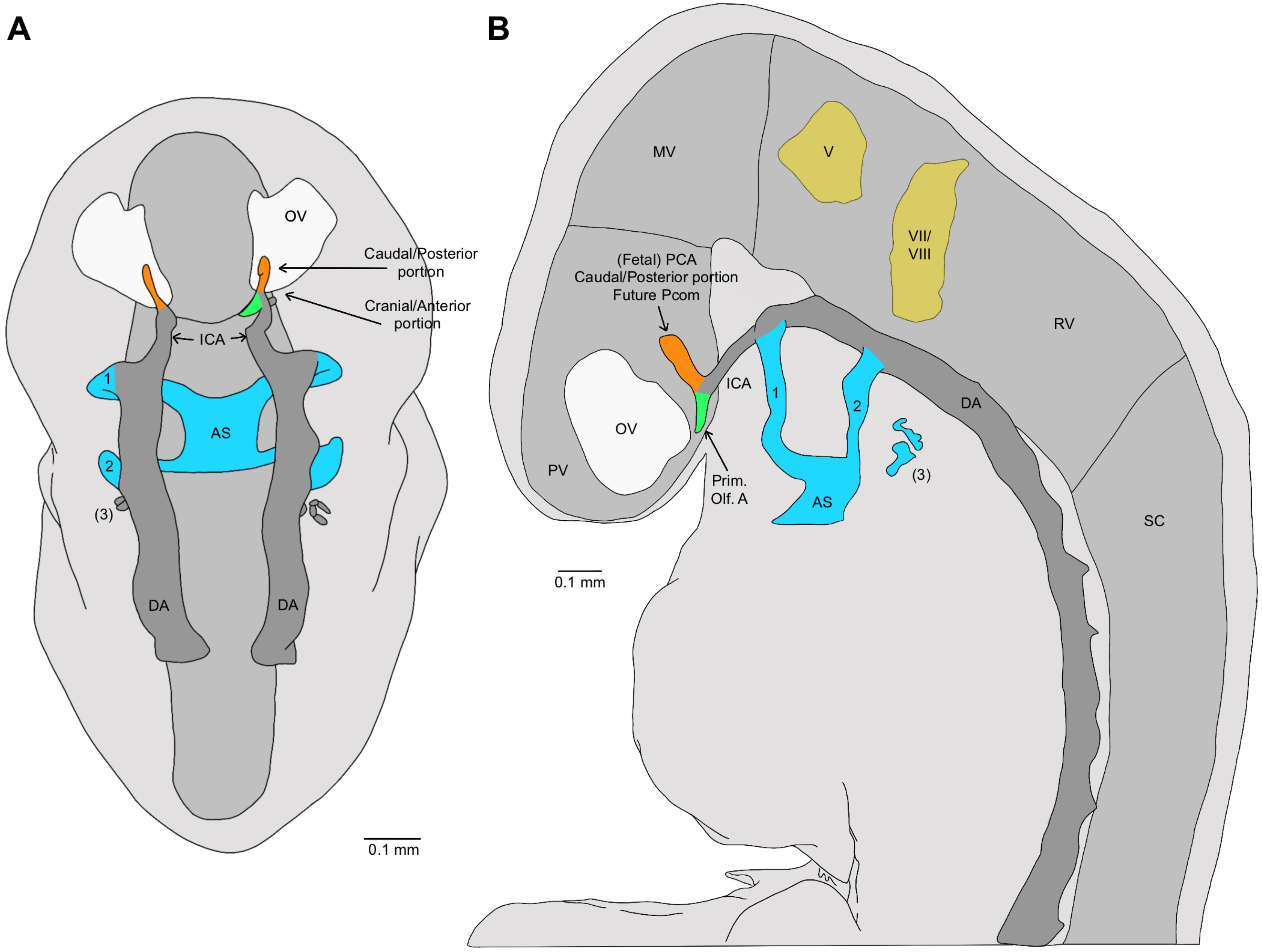

Carnegie stage 12 (26-30 days, 2.9 mm) specimen 8505A

Amsterdam UMC

ACTA Academic Centre for Dentistry Amsterdam

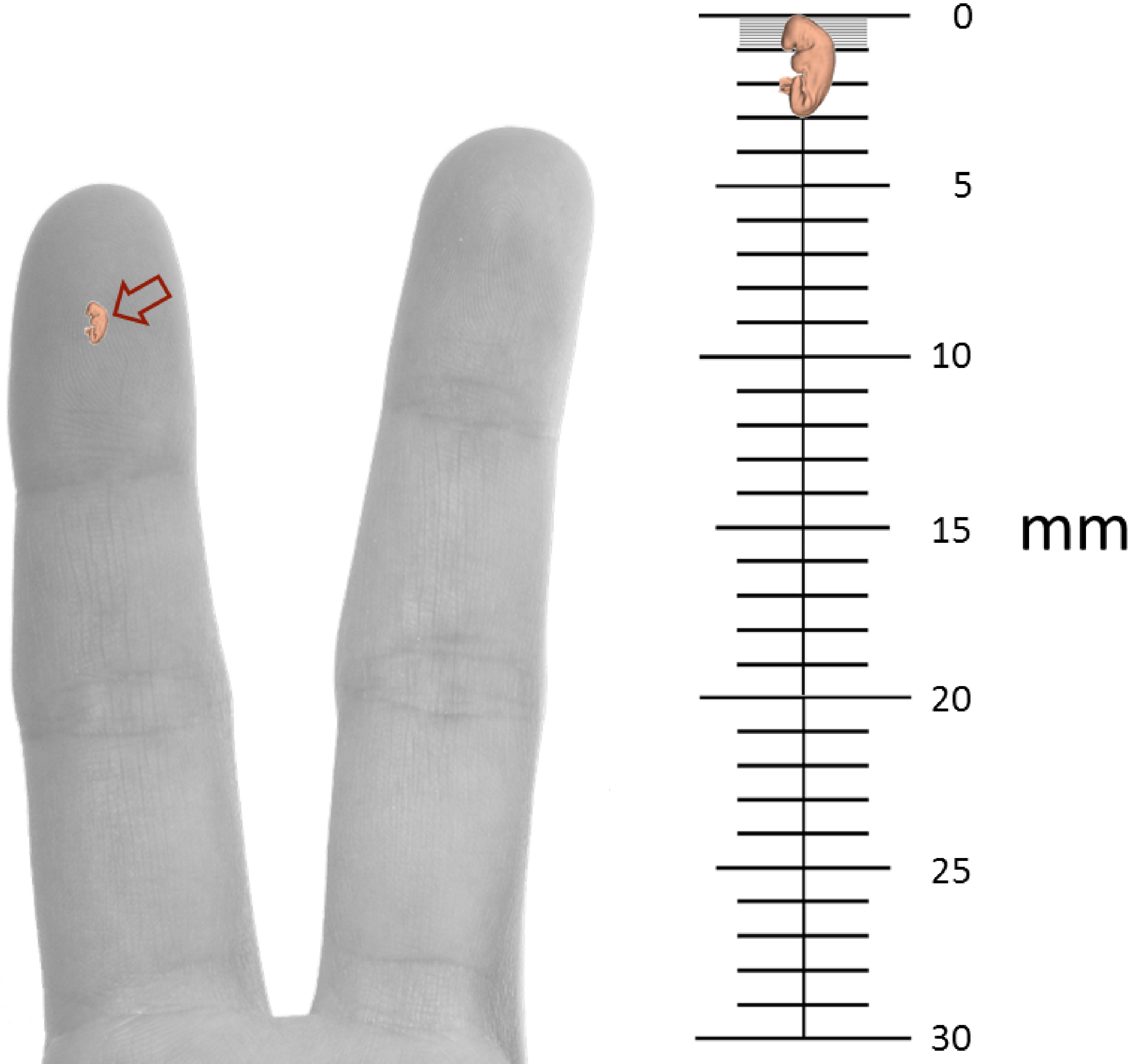

Supplement: Supplementary file 1 [file life-16-01153-s001.zip › Supplementary Material S1-Interactive 3D-PDF Models of Human Embryonic Intracranial Arterial Development (Carnegie Stages 11–23) .zip!/3DAtlas_CS12-8505A_NV_v1_3D.pdf]
