# Supplementary material for: Vascular Neuroembryology: The Genesis of the Intracranial Arterial System and the Circle of Willis
Source: Life (Basel). 2026 Jul 13;16(7):1153. doi: 10.3390/life16071153 (PMC13412907; doi:10.3390/life16071153)

Carnegie stage 14 (31-35 days, 5.5 mm) specimen 6520

Amsterdam UMC ACTA Academic Centre for Dentistry Amsterdam

All structures

Skin

Neural tube

Cranial nerves

Eye

Arteries all

Arteries left

Arteries right

Primitive olfactory arteries

Di/Mes-encephalic arteries

Basilar artery

Met/Myl-encephalic arteries

Vertebral arteries and branches

Carotid arteries

Pharyngeal arch arteries

Aorta and branches

Selected structure:

Use Adobe Reader with preferred settings (see below)

< Click here to enable 3D interaction >

Technical Note

View this PDF file in a recent version of Adobe Acrobat Reader: https://get.adobe.com/reader/

3D interaction is only possible on MS Windows or Mac OS. Javascript and playing of 3D content must be enabled by making these settings in Adobe Reader; *Menu, Preferences*:

1) In *JavaScript*
   - enable *Enable Acrobat JavaScript*
2) In *Multimedia & 3D*
   - enable *Enable playing of 3D content*
   - disable *Show 3D Orientation Axis*
   - *Optimization Scheme for Low Framerate: None*

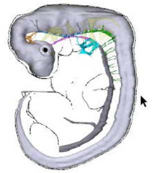

Overview

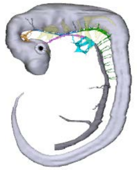

Neurovascular

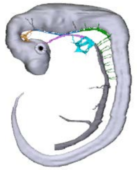

Vascular + Neural tube

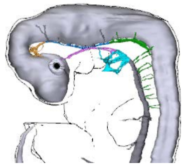

Zoom overview

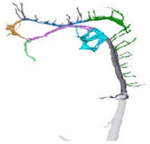

Lateral view arterial system

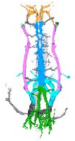

Cranial view arterial system

Carnegie stage 14 (31-35 days, 5.5 mm) specimen 6520

Amsterdam UMC

ACTA Academic Centre for Dentistry Amsterdam

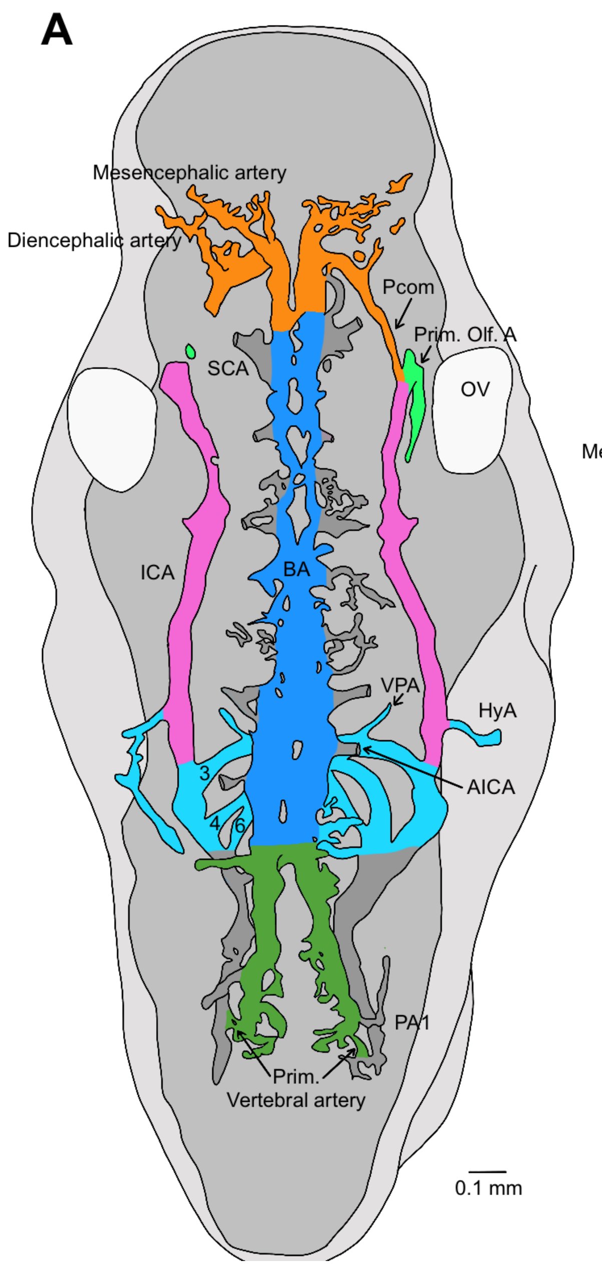

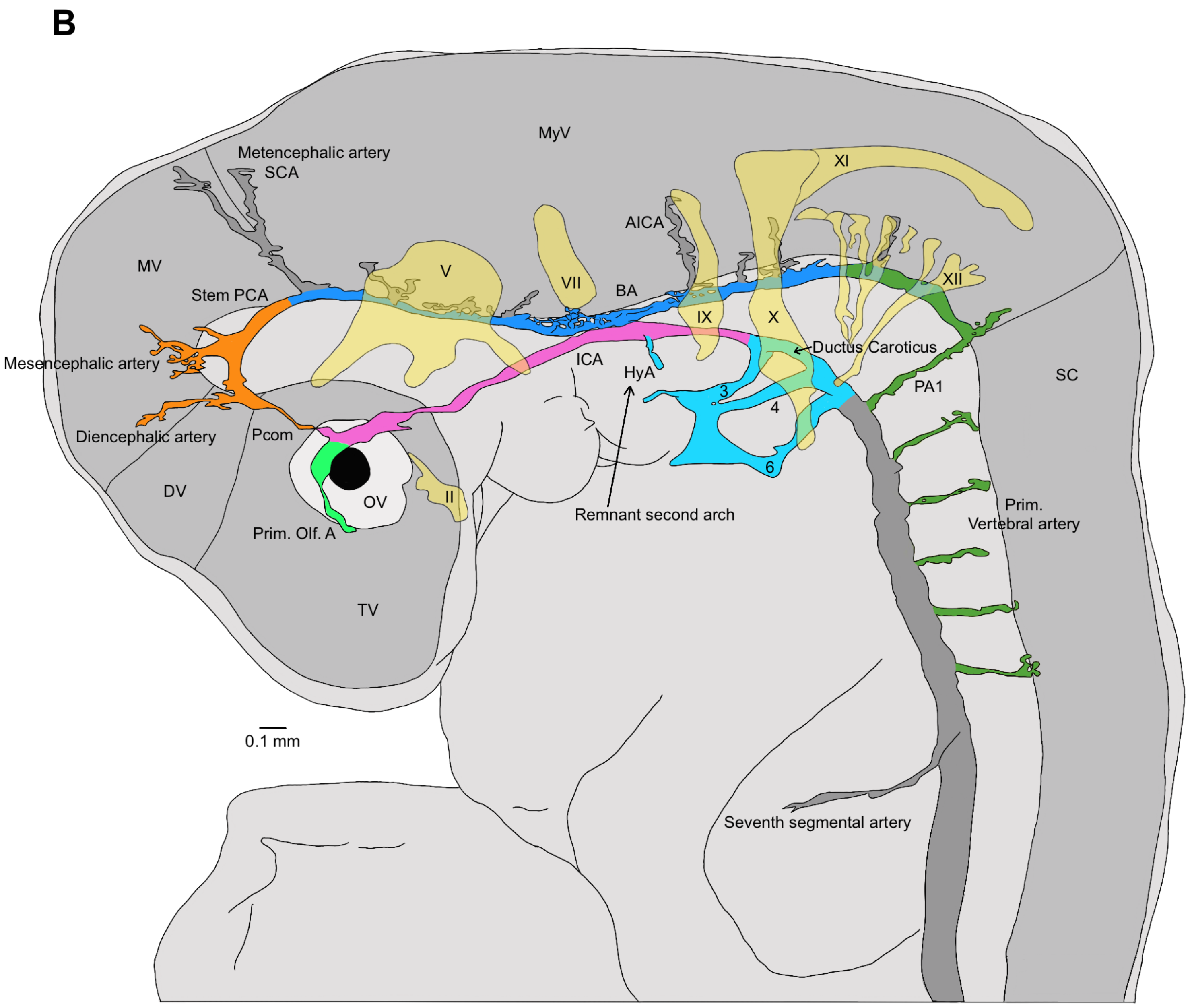

Carnegie stage 14 (31-35 days, 5.5 mm) specimen 6520

Amsterdam UMC

ACTA Academic Centre for Dentistry Amsterdam

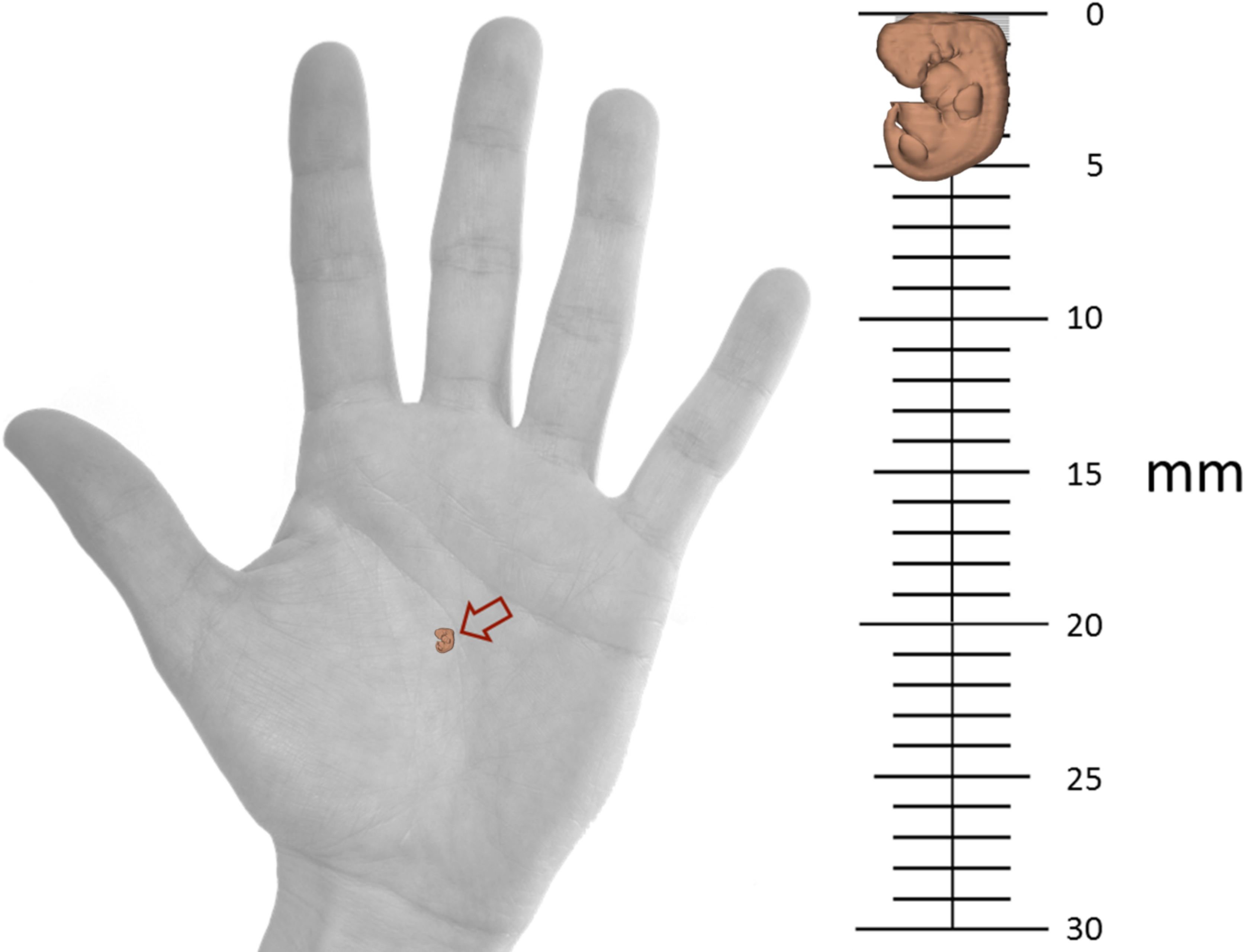

Supplement: Supplementary file 1 [file life-16-01153-s001.zip › Supplementary Material S1-Interactive 3D-PDF Models of Human Embryonic Intracranial Arterial Development (Carnegie Stages 11–23) .zip!/3DAtlas_CS14-6520_NV_v1_3D.pdf]
