# Supplementary material for: Vascular Neuroembryology: The Genesis of the Intracranial Arterial System and the Circle of Willis
Source: Life (Basel). 2026 Jul 13;16(7):1153. doi: 10.3390/life16071153 (PMC13412907; doi:10.3390/life16071153)

Carnegie stage 17 (42-44 days, 10.6 mm) specimen 6521

- All structures
- Skin
- Neural tube
- Cranial nerves
- Eye
- Arteries all
  - Arteries left
  - Arteries right
  - Telenchepalic arteries
  - Choroidal arteries
  - Di/Mes-encephalic arteries
  - Basilar artery
  - Met/Myl-encephalic arteries
  - Vertebral arteries and branches
  - Carotid arteries
  - Orbital arteries
  - Pharyngeal arch arteries
  - Aorta and branches

Selected structure:

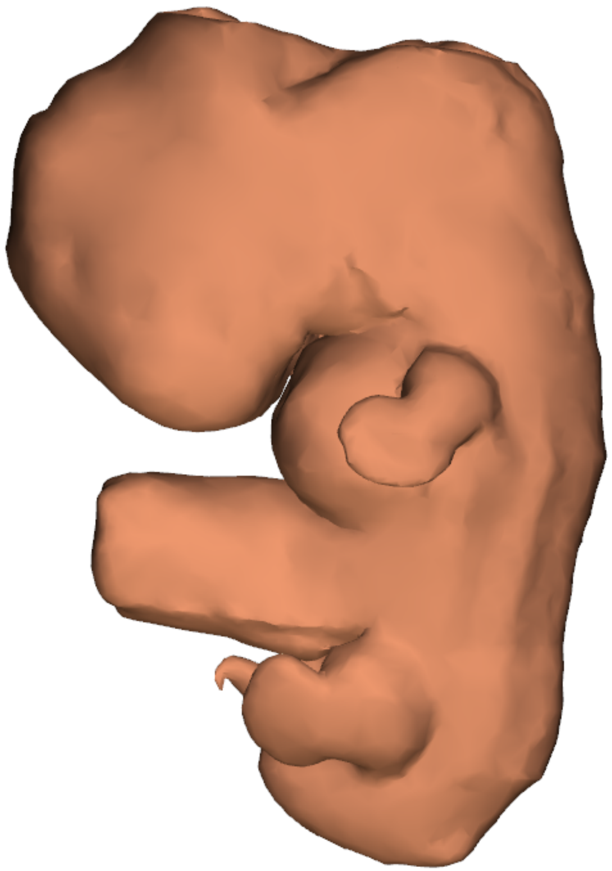

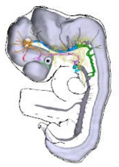

Overview

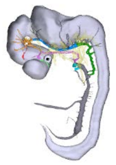

Neurovascular

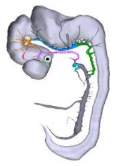

Vascular + Neural tube

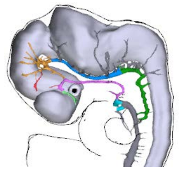

Zoom overview

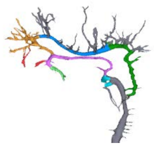

Lateral view arterial system

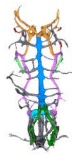

Cranial view arterial system

Carnegie stage 17 (42-44 days, 10.6 mm) specimen 6521

Amsterdam UMC

ACTA
Academic Centre for Dentistry Amsterdam

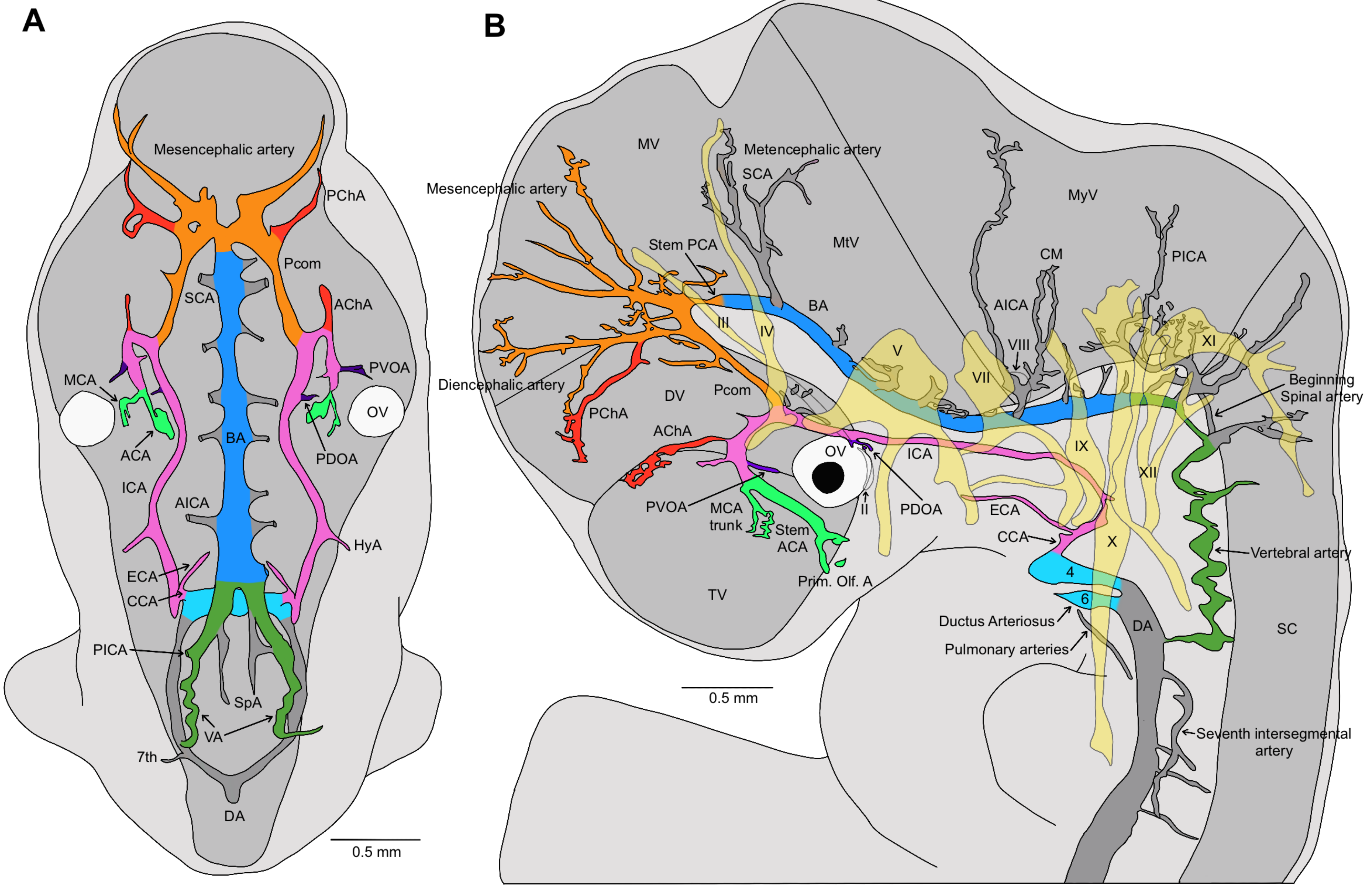

Carnegie stage 17 (42-44 days, 10.6 mm) specimen 6521

Amsterdam UMC

ACTA Academic Centre for Dentistry Amsterdam

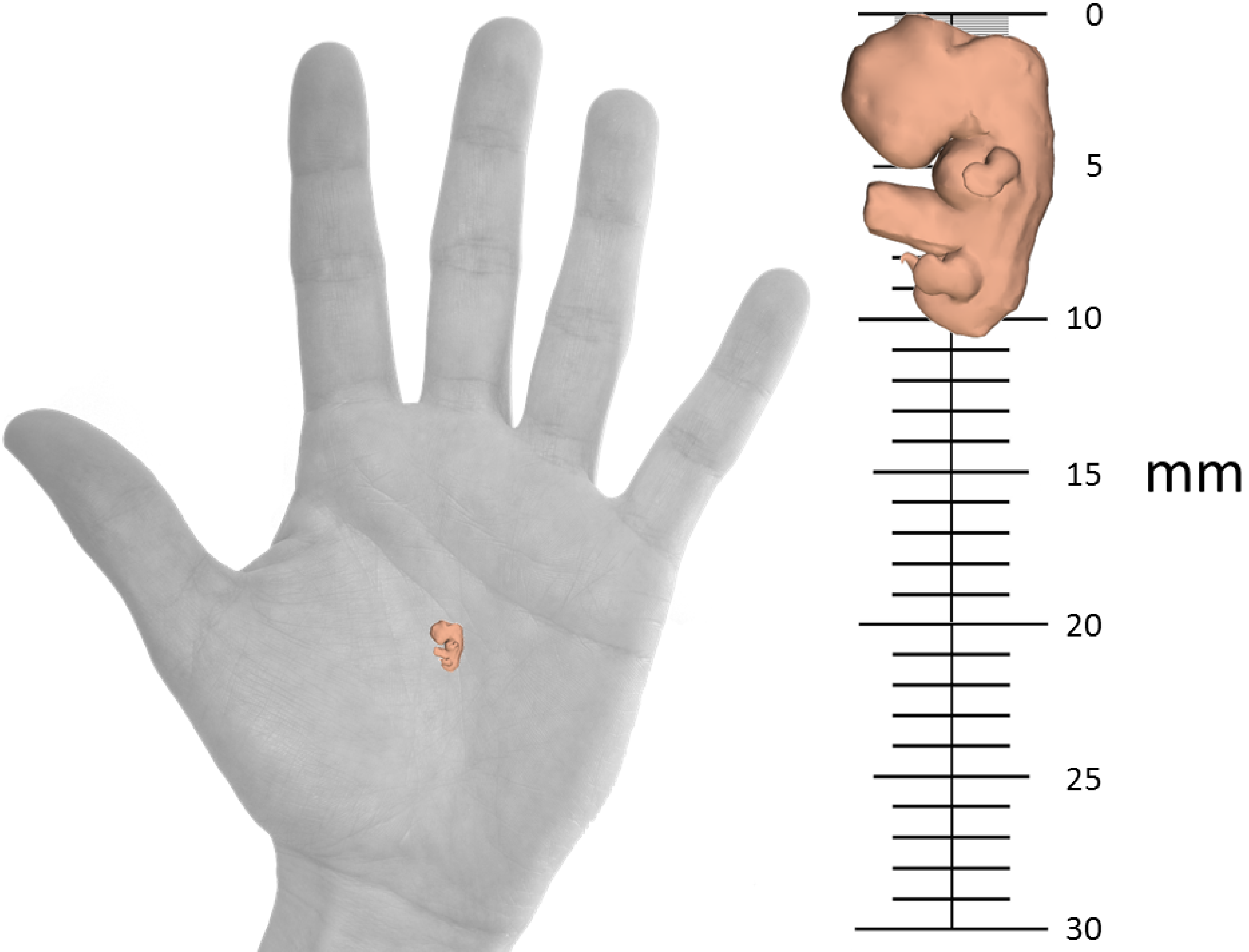

Supplement: Supplementary file 1 [file life-16-01153-s001.zip › Supplementary Material S1-Interactive 3D-PDF Models of Human Embryonic Intracranial Arterial Development (Carnegie Stages 11–23) .zip!/3DAtlas_CS17-6521_NV_v1_3D.pdf]
