# Supplementary material for: Vascular Neuroembryology: The Genesis of the Intracranial Arterial System and the Circle of Willis
Source: Life (Basel). 2026 Jul 13;16(7):1153. doi: 10.3390/life16071153 (PMC13412907; doi:10.3390/life16071153)

Carnegie stage 20 (51-53 days, 15.9 mm) specimen 462

Amsterdam UMC
ACTA Academic Centre for Dentistry Amsterdam

All structures
Skin
Neural tube
Cranial nerves
Eye
Arteries all
- Arteries left
- Arteries right
- Telenchepalic arteries
- Choroidal arteries
- Di/Mes-encephalic arteries
- Basilar artery
- Met/Myl-encephalic arteries
- Vertebral arteries and branches
- Carotid arteries
- Pharyngeal arch derivatives
- Aorta and branches

Selected structure:

Use Adobe Reader with preferred settings (see below)

< Click here to enable 3D interaction >

Technical Note

View this PDF file in a recent version of Adobe Acrobat Reader: https://get.adobe.com/reader/

3D interaction is only possible on MS Windows or Mac OS. Javascript and playing of 3D content must be enabled by making these settings in Adobe Reader; *Menu, Preferences*:

1) In *JavaScript*
   - enable *Enable Acrobat JavaScript*
2) In *Multimedia & 3D*
   - enable *Enable playing of 3D content*
   - disable *Show 3D Orientation Axis*
   - *Optimization Scheme for Low Framerate: None*

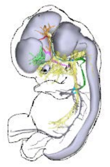
Overview

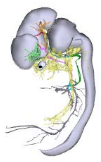
Neurovascular

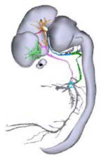
Vascular + Neural tube

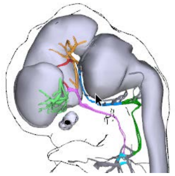
Zoom overview

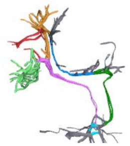
Lateral view arterial system

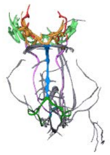
Cranial view arterial system

Carnegie stage 20 (51-53 days, 15.9 mm) specimen 462

Amsterdam UMC

ACTA Academic Centre for Dentistry Amsterdam

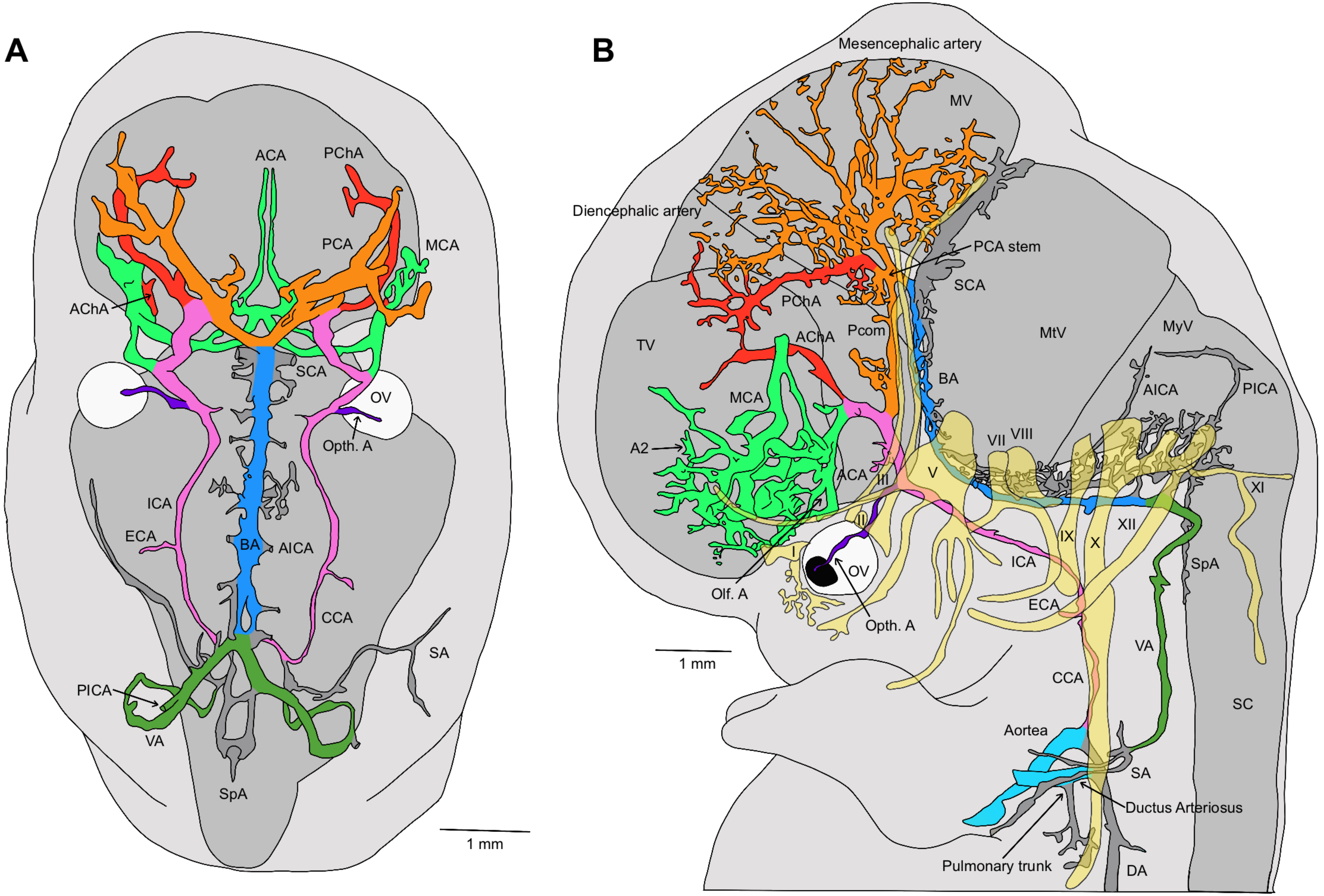

Carnegie stage 20 (51-53 days, 15.9 mm) specimen 462

Amsterdam UMC

ACTA Academic Centre for Dentistry Amsterdam

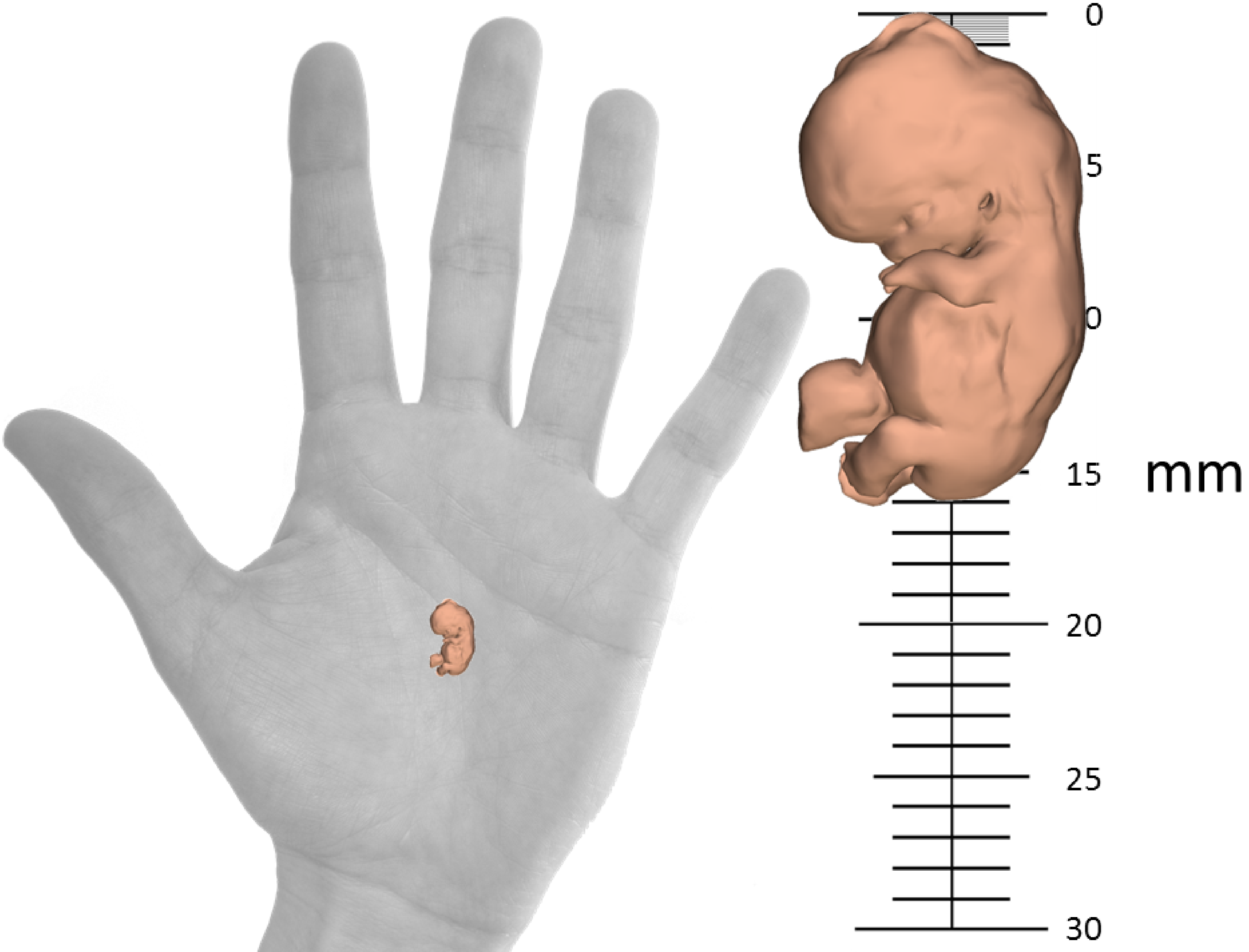

Supplement: Supplementary file 1 [file life-16-01153-s001.zip › Supplementary Material S1-Interactive 3D-PDF Models of Human Embryonic Intracranial Arterial Development (Carnegie Stages 11–23) .zip!/3DAtlas_CS20-462_NV_v1_3D.pdf]
